# No Evidence That Resting‐State Individual Alpha Frequency Represents a Mechanism Underlying Motion‐Position Illusions

**DOI:** 10.1111/ejn.70250

**Published:** 2025-11-02

**Authors:** Timothy Cottier, William Turner, Violet J. Chae, Alex O. Holcombe, Hinze Hogendoorn

**Affiliations:** ^1^ Melbourne School of Psychological Sciences University of Melbourne Melbourne Australia; ^2^ School of Psychology and Counselling Queensland University of Technology Kelvin Grove Queensland Australia; ^3^ The MARCS Institute for Brain, Behaviour, and Development Western Sydney University Westmead New South Wales Australia; ^4^ Department of Psychology Stanford University Stanford California USA; ^5^ School of Psychology University of Sydney Camperdown New South Wales Australia

**Keywords:** flash‐lag effect, Fröhlich effect, motion‐position illusions, resting‐state individual alpha frequency

## Abstract

Motion‐position illusions (MPIs) involve the position of an object being misperceived in the context of motion (i.e., when the object contains motion, is surrounded by motion or is moving). A popular MPI is the flash‐lag effect, where a static object briefly presented in spatiotemporal alignment with a moving object is perceived in a position behind the moving object. Recently, prior research has documented that there are stable individual differences in the magnitude of these illusions and possibly even their direction. To investigate the possible neural correlates of these individual differences, the present study explored whether a trait‐like component of brain activity, individual alpha frequency (IAF), could predict individual illusion magnitude. Previous reports have found some correlations between IAF and perceptual tasks. Participants (*N* = 61) viewed the flash‐lag effect (motion and luminance), Fröhlich effect, flash‐drag effect, flash‐grab effect, motion‐induced position shift, twinkle‐goes effect and the flash‐jump effect. In a separate session, 5 min of eyes‐closed resting state EEG data was recorded. Correlation analyses revealed no evidence for a correlation between IAF and the magnitude of any MPIs. Overall, these results suggest that IAF does not represent a mechanism underlying MPIs.

## Graphical abstract

There are noticeable individual differences in the perception of motion‐position illusions. In this study, we showed that individual differences in the perception of these illusions is not related to an individuals' resting‐state individual alpha frequency. This suggests that individual alpha frequency may not represent a mechanism underlying these illusions.

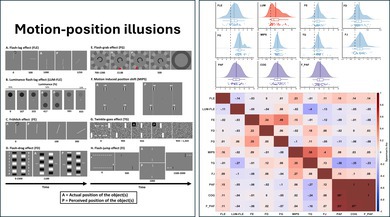

**Abbreviations**
BCa: bootstrapped bias‐corrected and accelerated
COG: centre of gravity
dva: degrees of visual angle
EEG: electroencephalography
FD: flash‐drag effect
FE: Fröhlich effect
FG: flash‐grab effect
FJ: flash‐jump effect
FLE: flash‐lag effect
F_PAF: FOOOF peak alpha frequency
IAF: individual alpha frequency
ICA: independent component analysis
LUM‐FLE: luminance flash‐lag effect
MIPS: motion‐induced position shift
MPIs: motion‐position illusions
MS: milliseconds
PAF: peak alpha frequency
PSE: point of subjective equality
SD: standard deviation
TG: twinkle‐goes effect

## 1 Background

Motion‐position illusions (MPIs) are a group of visual illusions, where the position of an object in the context of motion is incorrectly perceived. Typically, the object will contain internal motion, be surrounded by global motion or the object itself will be in motion. The mechanisms underlying these illusions are highly debated and limited neural correlates have yet to be identified. Recently, several studies have observed the presence of individual differences in the perception of MPIs (Cottier et al. 2023; Gauch and Kerzel 2008; Morrow and Samaha 2022). For some of these illusions, there is evidence that some participants consistently experience no illusory effect or the opposite of the expected effect. Individual differences often reflect differences in the optical and neural processes that mediate perception (Mollon et al. 2017). Therefore, by using an individual differences approach that takes these individual differences into consideration, we can better understand the mechanisms contributing to these illusions and visual perception in general. This research is fundamentally important for understanding the basis of individual differences in motion and position perception.

As our perception of the world appears continuous, visual perception is typically assumed to be a continuous process. However, several researchers have argued that visual perception might in fact be discrete (Herzog et al. 2020; Menétrey et al. 2022; VanRullen 2016; VanRullen and Koch 2003; White 2018). Similar to theories of discrete perception, discrete sampling is based upon the idea that visual input is sampled into discrete moments/windows, and perception results from a reconstruction of several discrete perceptual moments (Schneider 2018; Stroud 1967). Schneider (2018) proposed a model of discrete sampling to explain various properties of the flash‐lag effect (FLE), Fröhlich effect (FE) and related illusions.

**FIGURE 1:**
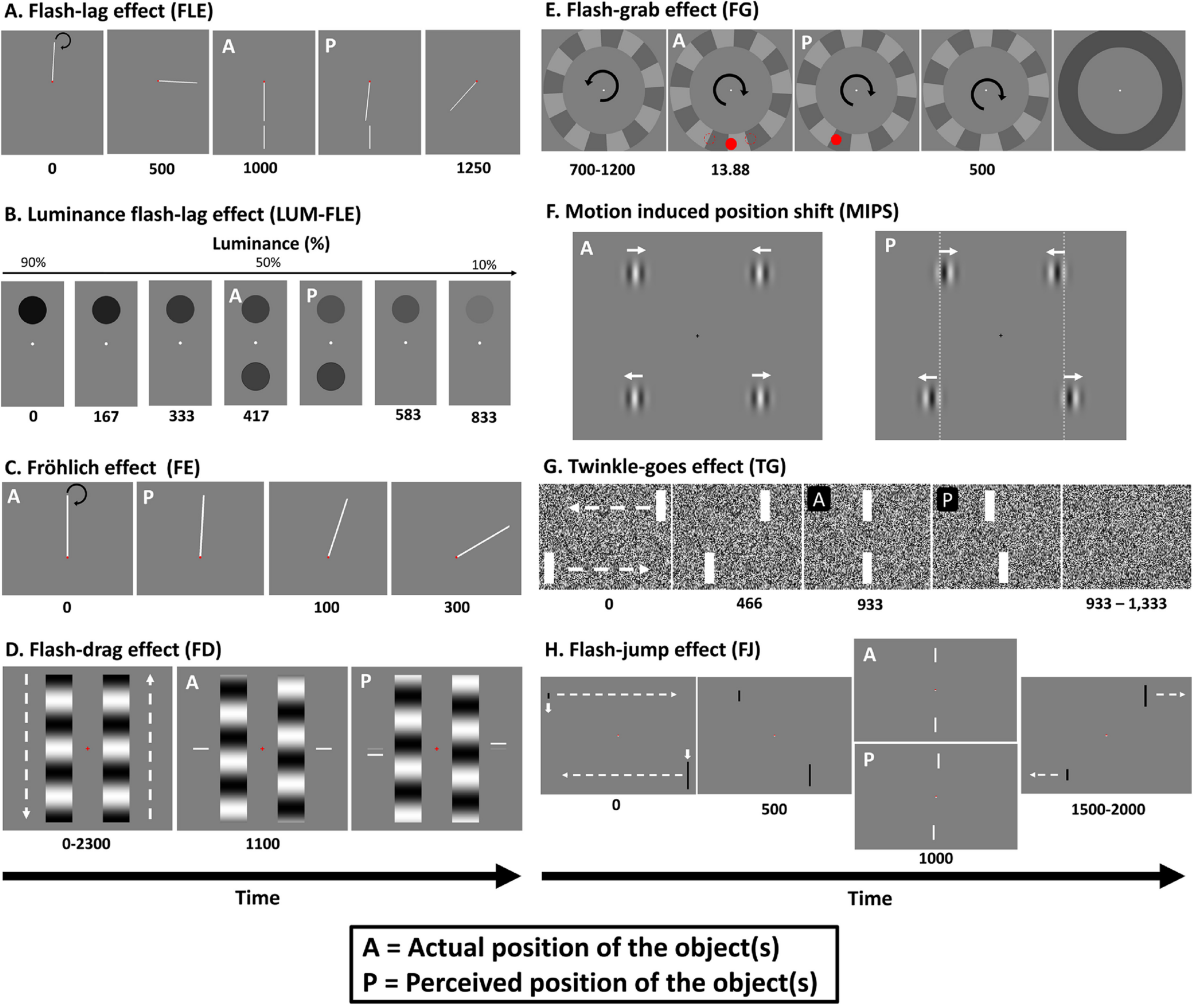
Motion‐position illusions. *Note:* Image and caption reproduced with permission from Cottier et al. (2023, 2), and consistent with their Creative Commons. ‘Stylized depictions of example trials for the eight motion‐position illusions used in this study. For all images, panels marked as “A” indicate the actual position of the object, and “P” indicates the perceived position of the object. (A) Flash‐lag effect (FLE): a rod rotates clockwise around the fixation point for 1250 ms. After 1 s, a stationary rod is briefly flashed in spatiotemporal alignment with the moving rod (actual). However, the moving rod is perceived mislocalized along its clockwise trajectory (perceived). (B) Luminance FLE (LUM‐FLE): the top circle decreases in luminance over 833 ms. Halfway through the trial, on the opposite side of the fixation point, a circle with identical instantaneous luminance is briefly presented (actual). Even though both circles have identical luminance values, the target circle is perceived further along its luminance trajectory and thus is perceived to be brighter than the flashed circle (perceived). (C) Fröhlich effect (FE): a rod rotates clockwise around the fixation point. When the rod initially appears, it is pointing straight up (actual), but it will be perceived in a position along its clockwise trajectory (perceived). (D) Flash‐drag effect (FD): two sinusoidal gratings move in opposite directions for 2300 ms. In this trial, the right grating is moving upward, while the left grating moves downward. After 1100 ms, two bars are flashed on the outside of each grating. While these bars are presented in vertical alignment (actual), they are perceived mislocalized in the direction of their nearest grating's motion (perceived). (E) Flash‐grab effect (FG): an annulus rotates counterclockwise for 800 ms, then reverses direction and rotates counterclockwise for 500 ms before turning grey. At the moment the annulus reverses direction, a red circle is flashed for 13.88 ms in one of three positions (the dotted red lines). After the annulus turns grey, participants report the perceived location of the target with a mouse click. In this trial, the red circle was presented at the bottom centre of the annulus (actual). However, this circle is perceived to be displaced in the reversal's direction of motion (perceived). (F) Motion‐induced position shift (MIPS): two pairs of vertically aligned gratings are presented (actual). The phase of the top gratings drifts toward the fixation point, while the phase of the bottom gratings drifts away from the fixation point. Even though the gratings are vertically aligned, they are perceived offset in their direction of motion (perceived). (G) Twinkle‐goes effect (TG): two bars translate toward one another for 933 ms. The top bar is moving right to left, and the bottom bar is moving left to right. When the bars are vertically aligned (actual), they disappear on a background of dynamic noise. The perceived offset positions of the two bars are shifted forward along their respective trajectories, such that they are seen as misaligned (perceived). (H) Flash‐jump effect (FJ): involves two bars moving toward each other and changing in height. In this trial, the top bar was moving left to right and increasing in height, while the bottom bar moved right to left while decreasing in height. When the two bars reach the centre of the screen and are physically aligned, they will be the same height and briefly become white (actual). This brief colour change is mislocalized further along the motion and growth trajectory of the bar and as such is perceived when the bar is a different size and not vertically aligned with the other bar (perceived)’.

The FLE (Figure 1A) involves briefly presenting a static object (the flash) in spatiotemporal alignment with a moving object (Nijhawan 1994). While the two objects are physically aligned in time and space, the moving object is perceived in a position further along its motion trajectory, and the flashed object is perceived to lag behind. According to Schneider (2018), the FLE occurs because a moving object continues to move throughout a perceptual moment and is perceived as its last position in a given moment. Conversely, on average, the flash will have occurred prior to the end of the moment. When the flash is experienced at the end of the moment in its veridical position, the moving object will have progressed further along its trajectory and will thus be experienced at a more advanced position. Schneider (2018) proposed that this discrete sampling and reconstruction process could correspond to alpha oscillations. However, this has yet to be explicitly tested.

Alpha oscillations (7–13 Hz) are one of the most prominent brain rhythms in human neural recordings (Klimesch 1999). Alpha oscillations predominantly occur over the occipital cortex and, thus, are likely to reflect the sensory aspects of visual perception (VanRullen 2016). Two studies have demonstrated how various features of alpha oscillations may influence the FLE, the most well‐known MPI (Chakravarthi and VanRullen 2012; Chota and VanRullen 2019). In 2012, Chakravarthi and VanRullen found a strong correlation between the FLE and pre‐stimulus occipital theta and alpha phase between 5 and 10 Hz (with a peak at 7 Hz), and between high‐alpha to low‐beta band post‐stimulus phase in the frontocentral electrodes (12–20 Hz). Consistent with these findings, Chota and VanRullen (2019) found that the FLE magnitude could be modulated by an entrainer oscillating at 10 Hz. These studies suggest that periodic alpha oscillations may modulate the perception of at least one MPI and thereby lend some support for the theory that discrete sampling underlies the FLE. However, these studies do not provide any insight into the extent to which alpha oscillations modulate perception of the broader class of MPIs, including the FE and flash‐jump effect (FJ), which the perceptual sampling account attempts to explain (Schneider 2018). It is important to conduct research investigating the mechanisms underlying multiple illusions, as most MPIs do not appear to share underlying neural processes (Cottier et al. 2023). Therefore, it is inappropriate for research to simply explore the mechanisms underlying a single illusion and generalise these findings to other illusions. Thus, while Schneider (2018) proposed that discrete sampling underlies multiple illusions, it might only be responsible for a single illusion.

If alpha oscillations contribute to the perception of MPIs, then alpha might predict individual differences in those illusions. Individual alpha frequency (IAF) is a trait‐like component of alpha, with high heritability (Smit et al. 2006), that is unique to each individual and stable over time, with excellent test–retest reliability (Grandy et al. 2013). IAF has been shown to correlate with general cognitive performance (Grandy et al. 2013), feature binding (Zhang et al. 2019) and spatial localisation (Howard et al. 2017).

Several studies have argued that IAF may index the temporal resolution of visual perception (for a review, see Samaha and Romei 2024). Samaha and Postle (2015) found that IAF is related to whether two flashes presented in close proximity are perceived separately or instead fuse and are perceived as a single flash. They found that participants with a faster IAF could perceive both flashes at a shorter interstimulus interval than those with a slower IAF. On this basis, they argued that IAF is related to the segregation and integration of incoming sensory information, with individuals with faster IAF more able to segregate the two flashes as distinct entities at shorter interstimulus intervals. These influential findings have been replicated by several researchers (for a review, see Samaha and Romei 2024), most recently by Deodato and Melcher (2024). These past studies thus provide solid evidence that IAF can reliably index individual differences in visual perception.

Empirical evidence has also emerged showing that IAF is related to the perception of illusions and motion. For example, IAF has been linked to the perception of the sound‐induced double flash illusion (Cecere et al. 2015), the bistable stream‐bounce display (Ronconi et al. 2023), the perceived frequency of the illusory jitter in the motion‐induced spatial conflict (Minami and Amano 2017), the flickering wheel illusion (Sokoliuk and VanRullen 2013), the spatial localisation of moving objects (Howard et al. 2017) and contrast detection abilities (Tarasi and Romei 2024). Overall, these studies suggest that IAF is related to individual differences in visual perception.

Regarding the FLE and FEs in particular, Morrow and Samaha (2022) argued that if discrete sampling at alpha was contributing to the flash‐lag and FEs, then the illusion magnitudes of these effects should correlate with one another. This is based upon Schneider's (2018) model, if one accepts that IAF indexes the duration of an individual's perceptual moment. However, Morrow and Samaha (2022) did not find a correlation between the Fröhlich and FLEs ( *r* _s_ = −0.008, 95% CI = [−0.41, 0.39]), suggesting that these illusions may not be predominantly caused by a shared underlying process. This finding could be a false negative, as their small sample size did not provide sufficient statistical power to detect weak to moderate effects. However, Cottier et al. (2023) also found that the correlation between the Fröhlich and FLEs was close to zero (*r*_s_ = 0.1, 95% BCa CI = [−0.144, 0.336]), despite high individual task reliability and a much larger sample size. However, neither study analysed EEG to measure participants' IAF and explore whether it correlated with individual illusions. Overall, the empirical evidence suggests that IAF is related to individual differences in visual perception, and aspects of alpha oscillations are related to the perception of the FLE (Chakravarthi and VanRullen 2012; Chota and VanRullen 2019). On this basis, we propose that IAF might be correlated with the perception of MPIs.

### 1.1 The Present Study

The present exploratory study assessed whether resting‐state IAF is related to the magnitude of eight MPIs. This study was an extension of Cottier et al. (2023). As such, we adopted an individual differences approach and had participants complete the FLE (Nijhawan 1994), luminance FLE (LUM‐FLE; Sheth et al. 2000), FE (Fröhlich 1924), flash‐drag effect (FD; Whitney and Cavanagh 2000), flash‐grab effect (FG; Cavanagh and Anstis 2013), motion‐induced position shift (MIPS; De Valois and De Valois 1991), twinkle‐goes effect (TG; Nakayama and Holcombe 2021) and flash‐jump effect (FJ; Cai and Schlag 2001). In order to calculate IAF, we also collected eyes‐open and eyes‐closed resting state EEG data in a separate experimental session. We hypothesised that IAF might correlate with the illusion magnitude of MPIs. This hypothesis was formulated on the basis that IAF has been correlated with a range of motion tasks and related illusions, and aspects of alpha appear to modulate the perception of the FLE illusions. To briefly foreshadow our results, we find no evidence for a relationship between resting‐state IAF and any of these illusions. This suggests that discrete sampling in the alpha range is unlikely to be responsible for MPIs. We also show that after correcting for multiple comparisons, we do not replicate the statistically significant correlations observed in Cottier et al. (2023); our correlation estimates are nevertheless similar. As such, we conduct an auxiliary analysis which provides updated estimates of the inter‐illusion correlation matrix by pooling the data from Cottier et al. (2023) and the present study.

## 2 Method

### 2.1 Participants

Cottier et al. (2023) found statistically significant correlations between certain MPIs of at least 0.37. Based on that, we used a correlation bivariate normal model from the exact test family (one‐tailed) in G*power (Version 3.1; Faul et al. 2009) to estimate a priori that we required a sample size of 59 participants to achieve 90% power to detect such effects (alpha level = 0.05). Therefore, 61 participants aged between 18 and 51 years (*M* = 25.6, SD [standard deviation] = 6.89; 44 females) were recruited from the University of Melbourne's paid research pool. Of these participants, 18 participated both in Cottier et al. (2023) and in a separate EEG study that recorded their resting‐state EEG. Participants were reimbursed $10/h for the behavioural component of the study and $15/h for the EEG component. All participants self‐reported as having correct or corrected to normal vision and no neurological deficits or disorders. Four participants reported being primarily left‐handed, the remaining participants were right‐handed. Some participants were excluded from analysis, which is discussed in detail in the pre‐processing section below. This study was approved by the University of Melbourne's Human Research Ethics committee, with separate approval provided for the illusion and EEG components (Illusion ID: 2022‐12816‐29275‐8, EEG ID: 2022‐12985‐29276‐6). Written informed consent was collected prior to participation. This research was conducted in accordance with University of Melbourne policies and Australian Government laws.

### 2.2 Apparatus

#### 2.2.1 Behavioural Experiment

Consistent with Cottier et al. (2023), stimuli were generated using PsychoPy (v2021.2.3; Peirce et al. 2019) and displayed upon a 24.5 in. ASUS PG258Q with a resolution of 1920 × 1080 pixels and a refresh rate of 144 Hz. The experiment ran off an HP EliteDesk 800 G3 TWR Desktop PC with an Nvidia GTX 1060 graphics card, with the Windows operating system. The monitor was gamma corrected using a Cambridge Research Systems ColorCal MKII (Cambridge Research Systems 2018). While participants completed the tasks, their head was stabilised with a SR Research chin and forehead rest placed approximately 50 cm from the monitor.

#### 2.2.2 EEG Experiment

Participants' electrophysiological activity was recorded using a 64‐channel BioSemi Active‐Two system, with a sampling rate of 512 Hz. Recordings were grounded using common mode sense and a driven right leg circuit; electrodes were attached to a standard 64‐electrode Biosemi EEG cap, with electrodes placed according to the international 10–20 system (Jasper 1958). An additional eight external electrodes were affixed to participants' skin: one on each mastoid, one above and below each eye, and one on the outer canthi of each eye. During recording, all electrode impedances were kept within ±50 μV.

### 2.3 Procedure

#### 2.3.1 Overall Procedure

Participants predominantly completed the behavioural and EEG components on separate days. At least two participants completed both sessions on the same day. Participants completed both components in a dimly lit room, while their head was placed upon a chinrest. The behavioural component took 2–2.5 h to complete, and the EEG component took 10 min to complete (excluding EEG setup). Electrophysiological data was not recorded while participants completed the behavioural task. The EEG data was often collected while participants were set up to participate in experiments unrelated to the present task.

#### 2.3.2 Overall Illusion Procedure

In a single session, participants were tested on eight MPIs. This involved participants completing eight experimental blocks in random order, with a separate block for each illusion (Figure 1). The eight illusions tested were: the flash‐lag effect (FLE; Nijhawan 1994), the luminance flash‐lag effect (LUM‐FLE; Sheth et al. 2000), the Fröhlich effect (Fröhlich, 1924), the flash‐drag effect (FD; Whitney and Cavanagh 2000), the flash‐grab effect (FG; Cavanagh and Anstis, 2013), the motion‐induced position shift (MIPS; De Valois and De Valois 1991), the twinkle‐goes effect (TG; Nakayama & Holcombe 2021), and the flash‐jump effect (FJ; Cai and Schlag 2001). The illusion procedure is identical to that used in Cottier et al. (2023). The only change made compared with Cottier et al. (2023) is that 16 practice trials were added to the beginning of the FE.

Prior to being assessed for each illusion, participants completed a Qualtrics survey, which checked their understanding of the experiment instructions, and then completed practice trials until they demonstrated sufficient understanding of each illusion (e.g., in the FLE, if the flash was 20° of polar angle in front of the moving target, we would make sure participants were reporting the flash as ahead). The understanding of participants was checked after each practice trial. Participants were asked to maintain fixation upon a fixation point (subtending approximately 0.3°–0.5° of visual angle) in the centre of a grey background. Breaks with no time limit were provided after each experiment block and halfway during each block. The luminance and motion FLE, the FE, FD and TG used a simple one‐up one‐down adaptive staircase procedure. For each of these illusions, several interleaved staircases were used, with separate staircases for direction of motion (e.g., left to right, or right to left) and position offset. The experiment code will be made available upon publication at https://osf.io/jxeu3/.

#### 2.3.3 Illusion Specific Procedure

This study uses the same tasks used in Cottier et al. (2023). As such, the methodological details were originally published in Cottier et al. (2023), but some details are reproduced here.

##### 2.3.3.1 Flash‐Lag Effect

A moving rod connected to the fixation point was displayed on the screen for a random duration of 1250, 1500, 1750 or 2000 ms. The rod rotated clockwise or counterclockwise at a speed of 180° of polar angle per second. In the last 250 ms of the rod's movement duration, a static rod was presented at a radius of 12.15 dva from the fixation dot for 49 ms. The edges of the static and moving rods were separated by 3.5 dva. The polar angle offset between the two rods was controlled by four randomly interleaved independent 1‐up, 1‐down adaptive staircases. There were two staircases for each direction of motion (clockwise and counterclockwise). For each direction of motion, one staircase began with the flash offset 28° of polar angle in the rod's direction of motion, and one began with the flash offset 28° of polar angle in the direction opposite of motion. The staircases started off with a stepsize of 4° of polar angle, reducing to 2° of polar angle after three reversals. Overall, participants completed 8 practice trials and 160 experimental trials (40 per staircase).

##### 2.3.3.2 Luminance Flash‐Lag Effect

Based upon the original paradigm by Sheth et al. (2000), a black target circle on a grey background (143 cd/m^2^) changed in luminance for 833 ms. For half the trials, the target either increased in luminance from 22 (black) to 132 cd/m^2^ (grey) or decreased in luminance from grey to black. Halfway through the task (416.4 ms), when the target had a luminance of 80 cd/m^2^, on the opposite side of the fixation cross to the target, a circle, ‘the flash’, was briefly presented for 14 ms. The centre of both circles was at an eccentricity of 3.5 dva from the fixation point. Both circles subtended 2.9 dva.

The contrast of the flash was set by four randomly interleaved independent 1‐up, 1‐down staircases. There were two staircases for each luminance change direction (increasing in brightness, decreasing in brightness). For each luminance change direction, there was one staircase that began with luminance contrast 100%, and another at 10%. The staircase step size was 5% of luminance contrast. Participants indicated on each trial which circle was darker.

##### 2.3.3.3 Fröhlich Effect

Connected to the fixation point was a black rod (10.5 dva long and 0.32 dva wide) that randomly rotated clockwise or counterclockwise around the fixation point for 604 ms. The rod rotated at a speed of 200° of polar angle per second. The starting position of the rod was controlled by four randomly interleaved independent 1‐up, 1‐down staircases. There was a total of 160 trials, with 40 trials per staircase. A break was provided halfway through the experiment. For each direction of rotation, there were two staircases. One staircase began with the rod offset 45° of polar angle from vertical in the direction of motion, and the other began with the rod offset opposite the direction of motion. The participants' task was to use the keyboard to indicate at the rod's onset, if the top of the rod was pointing to the left or right of vertical. After each trial, the onset position of the rod was changed by an increment that varied with every reversal in staircase direction (6°, 3°, 2° and 1° of polar angle).

##### 2.3.3.4 Flash‐Drag Effect

This flash‐drag paradigm was based upon the original paradigm by (Whitney and Cavanagh 2000). Two sinusoidally modulated greyscale liner gratings (20.5 dva high and 3.6 dva wide) with a spatial frequency of 0.15 cycles per dva were presented 3.85 dva to the left and right of a red fixation cross subtending 0.5 dva. The two gratings drifted vertically in opposite directions with a speed of 26.7 dva/s for 2300 ms. The gratings direction of motion reversed after every trial.

After the gratings had been in motion for either 1000, 1400, 1700 or 2000 ms, two horizontal white target rectangles (0.25 high and 2.05 dva wide) were presented at an eccentricity of 1.54 from the outer edges of the grating for 56 ms. The two rectangles were horizontally presented on opposite sides of the screen, such that one was on the left side of the screen, and another was on the right side of the screen. The vertical position of the flashes was always inverse. For example, if one flash was above the horizontal midline, the other was below the horizontal midline. The vertical position of the rectangles was controlled by four quasi‐randomly presented 1‐up, 1‐down staircases. There were two staircases for each direction of grating motion (up or down). One staircase began with the right flash being offset vertically 3.52 dva in the direction of motion, and another staircase began with the flash vertically offset 3.52 dva in the direction of opposition motion. The staircases were presented in pairs of opposite motion. To elaborate, a staircase with the right grating moving upward would be followed by a staircase with the right grating moving downward. Participants used a keyboard to report which flash was higher. The staircases adjusted the vertical position of the flash until they converged on the point of perceived equality. The vertical position was initially adjusted by 0.44 dva, then adjusted by 0.22 dva after four staircase reversals.

In total, participants completed 4 practice trials and 208 experimental trials, with a break provided halfway. During the practice trials, feedback was provided on whether participants correctly indicated which flash was higher.

##### 2.3.3.5 Flash‐Grab Effect

This section was copied from Cottier et al. (2023, 7), with permission from the authors. ‘Our flash‐grab paradigm was based on the paradigm used by Hogendoorn et al. (2015). A checkerboard‐textured black and white annulus with a radius of 17.8 dva randomly rotated clockwise or counterclockwise at 200 degrees of polar angle per second for a duration of 700 ms, 800 ms, 900 ms, 1,000 ms, 1,100 ms, or 1,200 ms, before reversing direction for 500 ms. At the center of the annulus was a stationary white fixation dot subtending 0.5 dva. When the reversal occurred, a red target circle subtending 3.2 dva was flashed for 14 ms. The red target circle was presented an equal number of times at three positions: the bottom center of the annulus, offset 20 degrees of polar angle to the left of center, or offset 20 degrees to the right of center. The center of the red target was presented at a radius of 14.15 dva from the fixation dot (i.e., centered on the width of the annulus). Participants completed 200 experimental trials: 30 trials for each of the six inducer durations and 20 catch trials in which no target was displayed to participants. Once the annulus had completed its movement, it became gray and participants used the mouse to report the perceived location of the red target on the annulus. When participants did not see the target, they reported this by clicking on the fixation point.’

##### 2.3.3.6 Motion‐Position Shift

This paradigm was based upon the paradigm by De Valois and De Valois (1991). Four vertically oriented Gabor patches were presented. Each Gabor had a diameter of 6.41 dva, a contrast of 90% and a spatial frequency of 0.46 cycles per dva. Two Gabor patches were vertically offset 9.61 dva above the fixation cross, and two were offset 9.61 dva vertically below. There were two to the left and two to the right of a black fixation cross. This fixation cross subtended 0.5 dva and was positioned in the centre of the screen. On each trial, the Gabors' horizontal positions were randomly chosen from one of three possible combinations of positions: (a) the top and bottom were vertically aligned, with all Gabors offset horizontally by 9.71 dva from fixation; (b) the top Gabors were offset horizontally from fixation by 12.16 dva, while the bottom Gabors were offset horizontally by 7.26 dva; and (c) the top Gabors were offset from fixation by 7.26 dva, while the bottom Gabors were offset away from fixation by 12.16 dva. The phase of each Gabor drifted horizontally within its static envelope at a speed of 4.3 dva/s, with each Gabor drifting in the opposite direction to its vertical partner. For example, the upper pair of Gabors could drift outward while the lower pair drifted inward or vice versa. All Gabors continued drifting throughout each trial, and participants used the keyboard to adjust the horizontal position of the two pairs of Gabors such that they were vertically aligned. Participants completed 60 trials, comprising 20 trials for each of the three different starting offsets. Prior to starting the experiment, participants practised the task (∼usually two to five trials) in front of the researcher.

##### 2.3.3.7 Twinkle‐Goes Effect

Based on the initial report of the illusion by Nakayama and Holcombe (2021), our twinkle‐goes paradigm used the same stimulus parameters as their first experiment. Stimuli were presented on a background of visual noise comprising squares (0.26 × 0.26 dva) with random luminance values. Stimuli consisted of two rectangles (2.9 dva wide × 7.7 dva high), presented diagonally above and below fixation, on opposite sides of the screen (left or right) at an initial eccentricity of 7.7 dva. Both rectangles translated horizontally toward the midline at a speed of 18.1 dva/s for a random duration between 800 and 1000 ms before disappearing. After the rectangles disappeared, the background noise remained on the screen for 400 ms before disappearing, and the screen went grey. On half of the 320 trials (the illusion condition), 80 ms before the rectangles disappeared, the background noise became dynamic by randomly and independently modulating the luminance values of each of the background noise squares. In the remaining trials (the static noise condition), the original static noise pattern was presented. In both conditions, the fixation point disappeared concurrently with the noise background, after which the screen remained grey until a key response was made.

On each trial, the disappearance location of the squares was controlled by one of eight randomly interleaved adaptive 1‐up, 1‐down staircases (40 trials per staircase). There were four staircases for each noise condition, two for each direction of motion (left to right or right to left). All staircases began with the squares disappearing out of vertical alignment, offset horizontally either 4.52 dva in the direction of motion or in the opposite direction. Staircases were used to converge on the point of perceived equality, adjusting the displacement between the squares with increments of 0.45 dva. Participants completed 332 trials total: 12 practice trials and 320 experimental trials.

##### 2.3.3.8 Flash‐Jump Effect

Inspired by the original report of Cai and Schlag (2001), our flash‐jump paradigm comprised a central fixation point and two vertical bars (0.36 dva wide), presented in diagonally opposite visual quadrants. The two bars began in a position with a horizontal eccentricity of 14.52 dva before horizontally translating toward the fixation point at a speed of 14.52 dva/s for 2000 ms. While the bars were moving, one bar grew in height while the other shrank in height. The heights changed at 4.32°/s. On approximately half of the trials (either 22 or 23 of the 45 experimental trials), the top bar grew in height while the bottom bar shrank in height, with the opposite occurring on the remaining half of the trials. Midway through the motion trajectory, while the bars were vertically aligned, both bars briefly flashed white for 14 ms.

The display repeated continuously while participants used a keyboard to adjust the height of one of the two bars, which we will call the target bar, until it was the same perceived height as the other bar at the instant that they both flashed white. The target bar was presented above fixation on half the trials and below fixation on the other half of the trials. The identity of the target bar was indicated to the participant by presenting either the top or the bottom half of the fixation point in red throughout the trial. Stimulus presentation on a trial was continuously repeated until participants pressed the ‘space’ key to indicate that they perceived the bars as being equal height when they flashed white.

Participants firstly completed 3 practice trials, before completing 48 randomly presented experimental trials. Of these 48 trials, 3 were attention checks that occurred every 15 trials. On a further third of trials, the target bar height at the moment of the flash was initially set to 1.93 dva taller than the other bar, and in the final third of trials, the target bar height at the moment of the flash was set to 1.93 dva shorter than the other bar. This variation in height was included to encourage participants to appropriately engage with the task, and seriously considered whether they needed to adjust the height of the bar on each trial. In our original publication, we observed that there was temporal imprecision in the timing of the flash's onset on a minority of trials. This could have resulted in the flash occurring when the bars were not the same height, as we intended. However, in both the present and previous study, we observed strong illusory effects. In fact, the previous publication showed they were reliable across two sessions. This inspires confidence that our paradigm was effective.

#### 2.3.4 Resting State EEG Procedure

The resting state session was organised into ten 60‐s trials, five trials for each condition (eyes‐open and eyes‐closed), sequentially alternating between conditions. All participants completed the trials in alternating order starting with an eyes‐open trial. Participants were instructed to stay still and relaxed throughout the recording, keeping their chin on the chinrest. During the eyes‐open trial, participants were told to fixate upon a white fixation dot in the centre of a grey background (RGB value = 128) and minimise blinking. During the eyes‐closed trial, participants were told to keep their eyes closed until they heard a beep signalling the start of the next trial. At the end of each trial, participants could take as long as they needed before pressing ‘space’ to proceed to the next trial. The start of each trial was indicated by an auditory beep played through the computer speakers. Resting state data collection was conducted by several researchers and could occur before or after participating in a separate EEG study. Four participants that completed Cottier et al. (2023) were brought back to complete just the resting state EEG component. The remaining participants provided resting state data during their participation for other EEG studies.

### 2.4 Analysis

#### 2.4.1 Behavioural Pre‐Processing

All data cleaning and analysis was conducted with MATLAB (v.R2023b; The MathWorks Inc. 2023). The analysis code will be made available upon publication at https://osf.io/jxeu3/. All behavioural data was cleaned and analysed using the analysis procedures described in Cottier et al. (2023). In brief, for each participant in each block, the magnitude of the associated illusion was estimated. For the five illusions that used 1‐up, 1‐down adaptive staircases (FLE, LUM‐FLE, FE, FD and TG), a point of subjective equality (PSE) was calculated for each staircase by averaging the final 20 trials for the FLE, LUM‐FLE and FD, and the final 10 trials for FE and TG due to fewer available trials. Within each direction of motion of the inducer or target (e.g., leftwards vs. rightwards motion, or clockwise vs. counterclockwise), a PSE for that direction was calculated by averaging across the PSEs for each staircase with that direction. For example, the PSE of the leftwards direction of motion would be the average of the PSEs for the two staircases containing leftwards motion. The illusion magnitude was then calculated as the average difference between the PSE for opposing directions of motion of the inducer or target (e.g., in the FLE (clockwise − counterclockwise) / 2). The average difference involves division by two, to ensure the illusion magnitude is not twice its true size.

The MIPS, FG and FJ did not use adaptive staircases, and for these illusions, the magnitude was calculated as the mean difference between the reported position and the physical position, within each direction of motion. In illusions utilizing staircases, participants were excluded if their staircases did not converge. The criteria for whether a staircase converged are discussed in each illusion‐specific subsection below. For participants that participated in Cottier et al. (2023) and completed two sessions, their illusion magnitude was calculated as the average magnitude of each of the two sessions.

##### 2.4.1.1 Flash‐Lag Effect

The FLE magnitude was calculated as the arc length distance in degrees of visual angle between the end of the target rod and the flash. This was done within each direction of motion (clockwise and counterclockwise), then averaged across motion directions. For this illusion, we considered staircases as not converged if the difference between the two staircases for a given motion direction (one initialized ahead and one initialized behind) was greater than 3.18° of visual angle (15° of polar angle). Six participants who completed a single session had staircases that failed to converge, and one participant who completed two sessions had staircases that did not converge. These participants were excluded. Of participants who completed two sessions, the staircases of three did not converge in one session, but did converge in the other. As such, their effect was calculated using the session where the staircases converged. Of the 61 participants who completed this illusion (18 completed two sessions), 7 participants were excluded from further analysis due to these staircase criteria. The final sample comprised 54 participants, 37 of whom completed a single session of illusions.

##### 2.4.1.2 Luminance Flash‐Lag Effect

The LUM‐FLE magnitude was calculated as the difference between the PSE of the luminance of the target circle and flashed circle, at the moment of flash onset. Staircases were considered not converged if, within any luminance change direction, the difference between the staircases with opposite initial values was greater than 30% luminance contrast. Applying this criterion, eight participants that completed a single session and one participant that completed two sessions were excluded from the analysis for this illusion. Three participants that completed two sessions of this illusion had staircases that did not converge in the first session but did converge in the second session. As a result, their LUM‐FLE effect was calculated using the data from the second session. Additionally, one participant was excluded because of a data‐saving error, where the csv was saved in a corrupt format. Overall, of the 61 participants that completed this illusion (18 completed two sessions), 10 participants were excluded. The final sample size comprised 51 participants, 34 of whom completed a single session of illusions.

##### 2.4.1.3 Fröhlich Effect

The FE was calculated as the arc length difference in degrees of visual angle between the starting position of the rod's trailing edge and the vertical meridian. Consistent with Cottier et al. (2023), participants were excluded if they pressed the same key for at least 80% of the trials in two or more staircases, or if their staircases did not converge. Staircases were considered to have not converged if, within a single motion direction, the difference between staircases with opposite starting values remained greater than 8.25 dva (45° of polar angle). Applying these exclusion criteria led to two participants who completed two sessions being excluded for having staircases that did not converge in either session. The final sample size comprised 59 participants, of whom 16 completed two sessions, 43 participants completed only a single session.

##### 2.4.1.4 Flash‐Drag Effect

On each trial, the FD was calculated as the vertical distance in degrees of visual angle between the PSE of the target rectangles and the central fixation point. The effect for each participant was calculated as half of the average difference between the PSEs in each direction (PSE for grating moving downwards − PSE for grating moving downwards / 2). Staircases were considered not converged if the final staircase values within a direction of motion differed by more than 3.5 dva. No participants failed the staircase exclusion criteria, so there were no exclusions, meaning that the final sample size for this illusion comprised 61 participants, 43 of whom completed a single session of illusions.

##### 2.4.1.5 Flash‐Grab Effect

The FG magnitude was operationalised as the arc length distance in degrees of visual angle between the target's position and the position reported by the participant. This was averaged across all trials within each reversal direction (clockwise and counterclockwise), then across reversals. Positive errors represent displacements in the direction of reversal motion. Participants were excluded if they failed more than 20% of the attention check trials or made invalid responses for more than 10% of the total trials (18 trials). Invalid responses were mouse responses not on the annulus on trials when the target was presented. Four participants who completed a single session were excluded for failing the attention check. One participant who completed two sessions was excluded for making too many invalid responses. Thus of the 61 participants who completed this illusion (18 completed two sessions), five participants were excluded. The final sample comprised 56 participants, of which 39 completed a single session of the illusions.

##### 2.4.1.6 Motion‐Induced Position Shift

The illusory effect was calculated as half of the average horizontal offset between upper and lower Gabors at the point that observers reported the two to be horizontally aligned. A trial was excluded as an outlier if the absolute magnitude of the effect was equal to or greater than 10° of visual angle. Of those that completed only a single session, two participants had a single trial removed, and two participants had two trialsremoved. Of the 18 participants that completed two sessions, seven participants had a single trial removed, and two participants had two trials removed. No participants were excluded from this illusion. However, due to technical issues accessing the laboratory, time constraints meant one participant was unable to complete this illusion. Therefore, the final sample size for this illusion comprised 60 participants; 42 of whom completed a single session of the illusions.

##### 2.4.1.7 Twinkle‐Goes Effect

The TG was operationalised as the difference between the PSE of the dynamic noise trials and the static noise trials. The PSE was calculated for each staircase averaged within direction and averaged across directions. The effect reflected half of the mean horizontal offset from vertical alignment at the point of perceptual alignment. Staircases were considered not converged if, within each direction of motion, staircases with opposite initial values had PSE differences greater than 1.48 dva. This criterion resulted in excluding three participants who completed a single session and one participant who completed two sessions. One participant who completed two sessions had staircases that did not converge in their first session but had staircases that all converged in their second session. As such, only their Session 2 data was used to calculate the effect. Overall, of the 61 participants, four were excluded, yielding 57 participants, 40 of whom completed a single session of the illusions and 17 that completed two sessions.

##### 2.4.1.8 Flash‐Jump Effect

The FJ was operationalised as half the average difference between the height of the target bar and the reference bar at the instantaneous moment of the flash. Positive values indicated an illusory shift in the direction of the size change (i.e., a growing bar was perceived as taller than veridical). To reduce the influence of premature responses, trials were considered outliers and excluded from the calculation if the magnitude on that trial was more than 3 SDs different from that participant's mean effect. Application of this rule led to one trial being excluded for six participants and two trials being excluded for one participant. Two participants who completed a single session failed all three attention checks and were excluded from further analysis. One participant that completed two sessions failed the attention checks in just a single session. As such, their illusion effect was calculated using their data from the one session where they passed the attention check. Furthermore, one participant (Participant 8) that completed two sessions had a single extra trial in their first session, and we are not sure why. Overall, of the 61 participants (18 completed two sessions), excluding two participants yielded 59 participants, 41 of whom completed only a single session of the illusions.

#### 2.4.2 EEG Pre‐Processing

EEG data was pre‐processed using the EEGLAB toolbox (Version 2024.0; Delorme and Makeig 2004) in MATLAB (Version R2023b; The MathWorks Inc. 2023). The raw data and channel spectra for the 19 parietal–occipital electrodes (P9, P7, P5, P3, P1, Pz, P2, P4, P6, P8, P10, PO7, PO3, POz, PO4, PO8, O1, Oz, O2) were manually inspected to identify and remove (and later interpolate, see below) channels that were flat‐lined or excessively noisy, and unlikely to contain signal. The data was then re‐referenced to the average signal of all the EEG electrodes, before being trimmed to contain only the parietal–occipital electrodes of interest. The data was downsampled to 256 Hz, the baseline (dc offset) was removed, and then the data was bandpass filtered using a 1 Hz high‐pass filter and a 40 Hz low‐pass filter. The continuous EEG data was then split into 10 distinct 62‐s epochs, from 1 s before the start of the trial to 61 s after the start of the trial. This epoch length was chosen to mitigate the effect of edge artefacts on the data (Cohen 2014). During data epoching, it was revealed that one participants had additional trials due to trigger sending issues with the analogue trigger box. This participant was retained for further analysis, as there was sufficient data to calculate eyes‐closed and eyes‐open IAF.

To clean the data, independent component analysis (ICA) was conducted using the infomax algorithm implemented using the extended runica function in EEGLab. The ICLabel classifier was used to automatically label the ICA components and automatically reject components that had a 90% or greater probability of being a muscle, eye or heart artefact (Pion‐Tonachini et al. 2019). Following ICA, the spherical spline method (Perrin et al. 1989) was used to interpolate removed channels. This resulted in a single channel being interpolated for eight participants and two channels being interpolated for three participants. The epochs were then trimmed to only contain the 60 s from the beginning of the trial.

#### 2.4.3 Calculating IAF

Individual alpha frequency (IAF) was primarily calculated using the automated algorithm developed by Corcoran et al. (2018). To ensure that aperiodic activity was not biasing our results, we also calculated IAF with the aperiodic component removed (F_PAF).

##### 2.4.3.1 Corcoran's Automated IAF Algorithm

Corcoran et al. (2018) provided an automated IAF algorithm that calculates two measures of IAF: peak‐alpha frequency and centre of gravity. The algorithm estimates the power spectral density using the MATLAB implementation (*pwelch.m*) of Welch’s modified periodogram method (Welch 1967). Then, a Savitzky‐Golay curve fitting method with a frame‐width of 11, and a polynomial order of 5, was used within the frequency window of 7‐13Hz to smooth the power spectral density output before estimating the peak alpha frequency and the centre of gravity. The peak alpha frequency is the frequency within the alpha band exhibiting the largest amplitude (Tarasi and Romei 2024). The centre of gravity computes a weighted average of the power within the alpha band, representing the average activity of alpha oscillations (Goljahani et al. 2012). The centre of gravity is a good measure of IAF when there are multiple alpha peaks or no alpha peak present in the EEG spectra, making it difficult to compute a distinct peak alpha frequency (Corcoran et al. 2018; Goljahani et al. 2012). Per the recommendations of Corcoran et al. (2018), in this study we report both measures of IAF.

For each measure of IAF and for each participant, we required an estimate of the measure of IAF for at least nine channels before averaging across channels. Using this criterion, for the eyes‐open condition, there were 17 participants for whom PAF could not be estimated, and 12 participants whose COG could not be estimated. In the eyes‐closed condition, for all participants (*N* = 61), COG and PAF could be estimated. Because reliable IAF estimates were not possible in the eyes‐open condition, we restricted our analyses exclusively to the data from the eyes‐closed condition. This is consistent with the fact that eyes‐closed data is often preferred due to its greater test–retest reliability (Grandy et al. 2013). Within our eyes‐closed condition, there was a strong significant positive correlation between PAF and COG (*r*_s_ = 0.95, *p* < 0.001), showing strong inter‐measure reliability between the two IAF estimates.

##### 2.4.3.2 Fitting Oscillations One Over F (FOOOF) IAF

MNE‐Python (Version 1.3.1) was used to load the pre‐processed Matlab files into Python. The data files were trimmed to contain only the five 60‐s epochs from the eyes‐closed condition. Welch's method (*fmin* = 1, *fmax* = 40) was used to extract the power spectral density estimates for each of the 19 parietal–occipital channels and for a subset of three electrodes (O1, Oz, O2). A global power spectral density estimate was created by averaging across the estimate for each channel. The FOOOF algorithm was implemented with its accompanying Python module (Version 1; Donoghue et al. 2020) to extract the aperiodic and periodic components of the global power spectral density. A FOOOF model with a fixed aperiodic mode was fit to each trial on frequencies between 2 and 20 Hz to identify a single peak (*max_n_peaks =* 1) within the alpha band (7–13 Hz) with a peak width between 0.5 and 12 Hz. We excluded the alpha peak estimates for trials with bad model estimates. The criterion for a bad model fit was the r squared being less than 0.6. After these exclusions, when the model was fit to data from the 19 parietal–occipital electrodes, five peaks were identified for 55 participants, four peaks were identified for two participants, three peaks were identified for two participants, two peaks were identified for one participant, one peak was identified for one participant. When the model was fit on a subset of parietal–occipital electrodes (O1, Oz, O2), five peaks were identified for 51 participants, four peaks were found for five participants, three peaks were found for three participants, and one peak and no peak were each found for a single participant. For each participant, IAF was operationalised as the PAF, the peak with the largest periodic power. The peak periodic alpha frequency calculated using the FOOOF algorithm is referred to as FOOOF PAF (F_PAF).

### 2.5 Statistical Inference

The histograms for each illusion and measure of IAF indicated that the data was not normally distributed (Figure S1). This was confirmed by statistically significant Kolmogorov–Smirnov tests (Table S1) with *p <* 0.001. As such, consistent with our previous publication (Cottier et al. 2023), non‐parametric statistical analyses were conducted, and 95% confidence intervals were calculated with bias‐corrected and accelerated (BCa) bootstrapping (*N* = 1000; Efron and Tibshriani, 1994). Spearman's Rho was used for the correlation analyses. Correlation estimates will always be attenuated by measurement noise (Mollon et al. 2017; Spearman 1987). As such, to correct for this measurement error and get a ‘true’ estimate of the correlations between IAF and the illusions, we calculated disattenuated correlations using Spearman (1987)’s formula (see also Cottier et al., 2023). Disattenuated correlations are reported alongside the regular ‘attenuated’ correlations. However, we will not interpret the disattenuated correlations, as they are simply provided as an estimate of the true effect, and are not intended for inference (Hedge et al. 2018). To calculate the disattenuated correlations, we used the test‐retest reliabilities for the illusions published by Cottier et al. (2023), and the test‐retest reliability for Grandy et al. (2013)’s eyes‐closed young IAF control group (0.87). The colours of all correlation matrices presented in this manuscript and the supplementary materials used the colorCET (‘D1A’) colourmap, which was implemented using the MATLAB “colorcet” Function (Kovesi, n.d.; Kovesi, 2015).

## 3 Results

### 3.1 Descriptive Statistics

**FIGURE 2:**
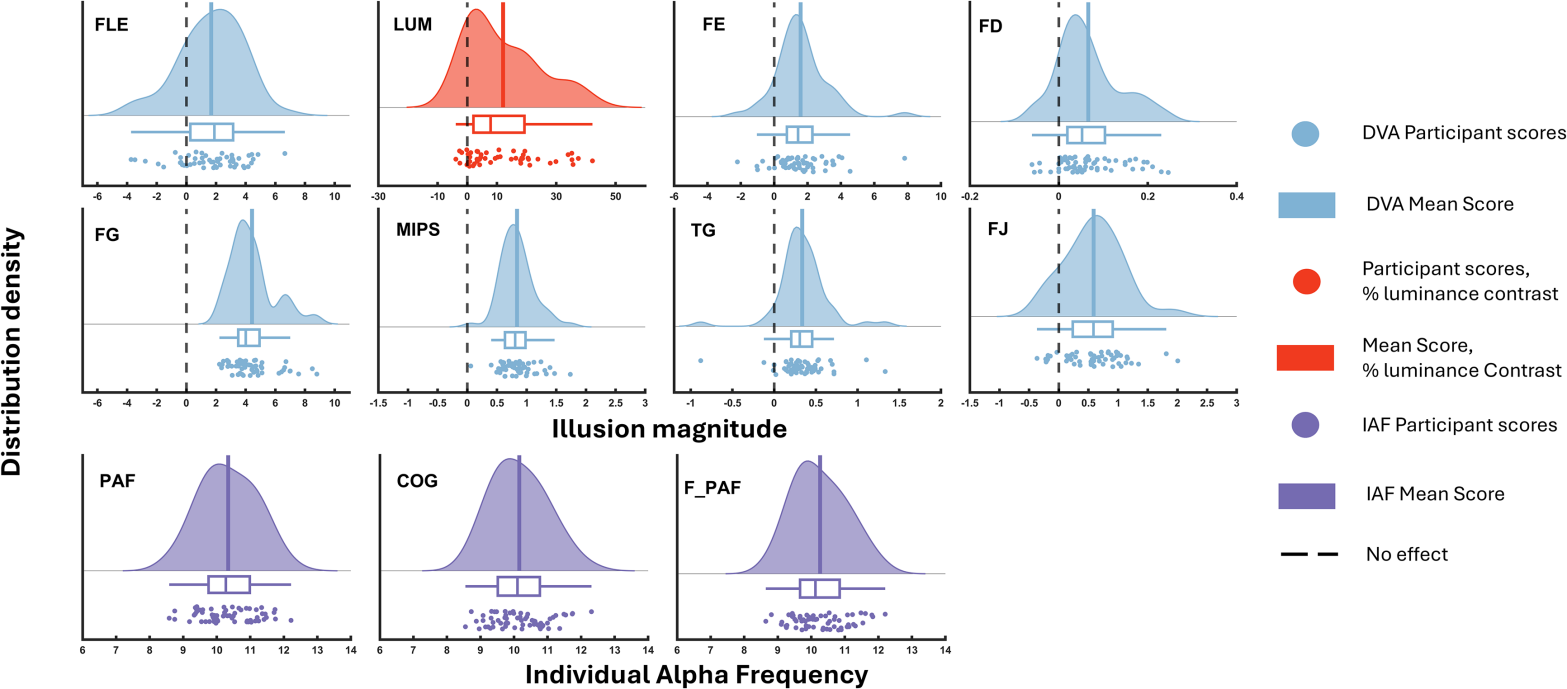
Raincloud plots for each illusion and the three individual alpha frequency (IAF) estimates. *Note:* Raincloud plots were created with Allen and colleagues (2019) MATLAB function. LUM shows luminance flash‐lag effect (LUM‐FLE). Blue colours show illusory magnitude measured in degrees of visual angle (dva), red colours show illusory effect measured in % of luminance contrast, and purple colours show measures of IAF. The dashed black line shows the point corresponding to no illusory effect, with positive values representing an illusory effect in the expected direction. Boxplots show the interquartile range and median. The distributions show an estimated probability density distribution created using MATLAB's ksdensity function with the mean marked with the solid vertical line. All raincloud plots colours were derived from Cynthia Brewer’s ColorBrewer color schemes (see: Harrower & Brewer, 2003). The colours for the LUM‐FLE and IAF raincloud plots were rgb values obtained from the ColorBrewer website ‐ https://colorbrewer2.org/ (Brewer et al., 2013). The colour or the remaining illusion raincloud plots was taken from colorbrewer Set3 and implemented using Lowe’s (2025) cbrewer2 MATLAB function.

**FIGURE 3:**
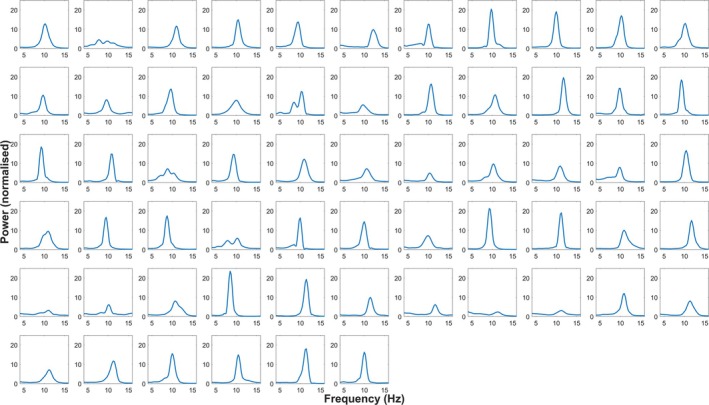
The Q‐weighted power spectral density estimate for each participant. *Note:* For each participant, the power spectrum was averaged across the power spectra for each channel. All participants showed a peak in the alpha band (7–13 Hz). These estimates were obtained and plotted using code published by Corcoran et al. (2018).

Figure 2 shows raincloud plots that provide the distribution, raw scores, range, median and interquartile range for each illusion. These were created using Allen et al. (2019) MATLAB function. Overall, illusory effect magnitudes were qualitatively similar to observations in Cottier et al. (2023) (Table 1). Inspection of the raincloud plots (Figure 2) suggests that there might be individual differences present in the magnitude of each illusion and in the measures of IAF. The mean PAF was 10.34 Hz (range = 8.58–12.21; SD = 0.83 Hz), the mean COG was 10.16 Hz (range = 8.55–12.31; SD = 0.83 Hz), and the mean F_PAF was 10.26 Hz (range = 8.64–12.20; SD = 0.81 Hz). Inspection of the power spectra (Figure 3) confirms that a peak in the alpha band was present for all participants. PAF and COG were strongly correlated (*r*_s_ = 0.95, *p* < 0.001, 95% BCa CI = [0.90, 0.98]), as was PAF and F_PAF (*r*_s_ = 0.92, *p* < 0.001, 95% BCa CI = [0.85, 0.96]) and COG and F_PAF (*r*_s_ = 0.96, *p* < 0.001, 95% BCa CI = [0.92, 0.98]).

**TABLE 1:** Mean and standard deviation for each illusion's magnitude.

Illusions	Unit of measurement	*M* (SD)	
		Present study	Cottier et al. (2023)
Flash‐lag effect (FLE)	Degree of visual angle	1.68 (2.14)	1.70 (1.78)
Luminance flash‐lag (LUM‐FLE)	% Luminance contrast	12 (12)	12 (13)
Fröhlich effect (FE)	Degree of visual angle	1.58 (1.53)	1.2 (1.36)
Flash‐drag effect (FD)	Degree of visual angle	0.07 (0.07)	0.06 (0.07)
Flash‐grab effect (FG)	Degree of visual angle	4.42 (1.51)	4.34 (1.65)
Motion‐induced position shift (MIPS)	Degree of visual angle	0.84 (0.28)	0.73 (0.25)
Twinkle‐goes effect (TG)	Degree of visual angle	0.34 (0.29)	0.38 (0.26)
Flash‐jump effect (FJ)	Degree of visual angle	0.59 (0.49)	0.44 (0.40)

*Note:* The table shows data for the present study and for Cottier et al. (2023).

### 3.2 Correlation Analyses

**FIGURE 4:**
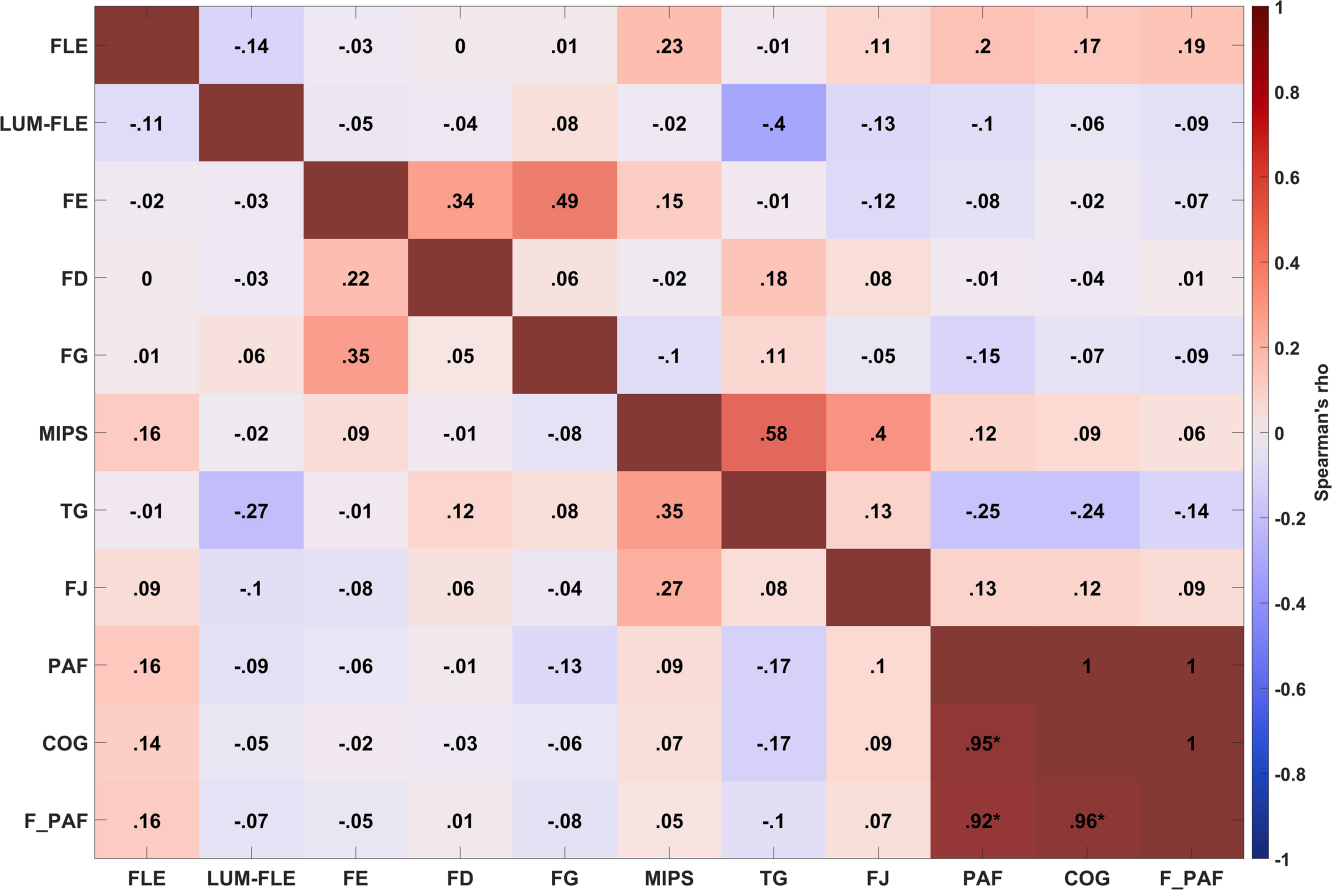
Correlations of the illusions with each other and with the measures of IAF. *Note:* IAF was measured with peak alpha frequency (PAF), centre of gravity (COG) and the FOOOF PAF (F_PAF) with the aperiodic element removed using the FOOOF algorithm. The disattenuated correlations are presented above the red diagonal, and the raw correlations are presented below the diagonal. Statistically significant (*p* < 0.01) correlations are marked with an asterisk, with the *p*‐values presented in Table S2. None of the illusions were significantly correlated with the IAF measures. See the Supporting Information for alternative analyses (which also did not yield significant correlations between the illusions and IAF).

We calculated Spearman's Rho correlation coefficients to explore whether individual differences in illusion magnitude were related to participants' IAF estimates (Figure 4). Scatterplots of the relationship between illusions and the measures of IAF are presented in Figures S2–S11. BCa bootstrapped (*N* = 1000) confidence intervals are presented in Table 2. Bonferroni–Holm correction (for 55 comparisons; 8 illusions and 3 measures of IAF) was used to control the family‐wise error rate for multiple comparisons (Holm 1979). The uncorrected *p*‐values for the correlation analyses are presented in Table S2. As shown in Figure 4, after Bonferroni–Holm correction, we observed no statistically significant correlations between any of the illusions or between any illusions and the measures of IAF. The only statistically significant correlations we observed were between our measures of IAF (PAF and COG, PAF and F_PAF, COG and F_PAF). However, prior to Bonferroni–Holm correction, statistically significant correlations were observed between the FE and FG (*r*_s_ = 0.35, *p* = 0.009, 95% BCa CI = [0.05, 0.57]), and the twinkle‐goes and MIPS (*r*_s_ = 0.35, *p =* 0.007, 95% BCa CI = [0.07, 0.57]). The former was not significant after correction for multiple comparisons in the data of Cottier et al. (2023), but the latter was. All confidence intervals contained zero, indicating no strong evidence for a relationship between IAF and the illusions.

**TABLE 2:** Confidence intervals for correlations of the illusions with each other and with the measures of IAF.

All Parietal‐Occipital electrodes											
Illusions	FLE	Lum‐FLE	Fröhlich	FD	FG	MIPS	TG	FJ	PAF	COG	F_PAF
**FLE**		[‐0.43, 0.18]	[‐0.34, 0.28]	[‐0.35, 0.16]	[‐0.31, 0.31]	[‐0.13, 0.44]	[‐0.27, 0.32]	[‐0.11, 0.45]	[‐0.02, 0.51]	[‐0.07, 0.49]	[‐0.06, 0.48]
**Lum‐FLE**	[‐0.40, 0.18]		[‐0.30, 0.30]	[‐0.30, 0.28]	[‐0.21, 0.34]	[‐0.31, 0.27]	[‐0.50, 0.04]	[‐0.38, 0.17]	[‐0.40, 0.24]	[‐0.36, 0.24]	[‐0.37, 0.25]
**Fröhlich**	[‐0.29, 0.29]	[‐0.31, 0.25]		[‐0.03, 0.44]	**[0.07, 0.59]**	[‐0.15, 0.31]	[‐0.28, 0.27]	[‐0.33, 0.20]	[‐0.31, 0.21]	[‐0.28, 0.25]	[‐0.29, 0.21]
**FD**	[‐0.28, 0.27]	[‐0.31, 0.25]	[‐0.03, 0.43]		[‐0.25, 0.26]	[‐0.28, 0.26]	[‐0.12, 0.44]	[‐0.17, 0.38]	[‐0.19, 0.33]	[‐0.18, 0.31]	[‐0.2, 0.33]
**FG**	[‐0.24, 0.31]	[‐0.21, 0.36]	**[0.05, 0.57]**	[‐0.22, 0.28]		[‐0.33, 0.19]	[‐0.19, 0.38]	[‐0.24, 0.23]	[‐0.36, 0.19]	[‐0.31, 0.27]	[‐0.33, 0.23]
**MIPS**	[‐0.14, 0.41]	[‐0.31, 0.29]	[‐0.19, 0.31]	[‐0.29, 0.28]	[‐0.33, 0.19]		**[0.06, 0.56]**	[‐0.02, 0.52]	[‐0.18, 0.34]	[‐0.18, 0.30]	[‐0.19, 0.28]
**TG**	[‐0.30, 0.30]	[‐0.50, 0.02]	[‐0.25, 0.27]	[‐0.13, 0.39]	[‐0.22, 0.34]	**[0.07, 0.57]**		[‐0.21, 0.30]	[‐0.45, 0.09]	[‐0.45, 0.09]	[‐0.38, 0.15]
**FJ**	[‐0.20, 0.37]	[‐0.35, 0.23]	[‐0.35, 0.20]	[‐0.23, 0.32]	[‐0.29, 0.21]	[‐0.02, 0.50]	[‐0.18, 0.34]		[‐0.22, 0.31]	[‐0.21, 0.30]	[‐0.24, 0.30]
**PAF**	[‐0.17, 0.43]	[‐0.40, 0.20]	[‐0.30, 0.20]	[‐0.26, 0.25]	[‐0.38, 0.14]	[‐0.18, 0.34]	[‐0.39, 0.10]	[‐0.17, 0.35]		**[0.90, 0.98]**	**[0.85, 0.96]**
**COG**	[‐0.18, 0.43]	[‐0.35, 0.23]	[‐0.28, 0.25]	[‐0.26, 0.21]	[‐0.31, 0.22]	[‐0.18, 0.32]	[‐0.40, 0.12]	[‐0.18, 0.34]	**[0.90, 0.98]**		**[0.92, 0.98]**
**F_PAF**	[‐0.17, 0.44]	[‐0.36, 0.21]	[‐0.31, 0.21]	[‐0.24, 0.26]	[‐0.33, 0.20]	[‐0.20, 0.29]	[‐0.34, 0.18]	[‐0.19, 0.32]	**[0.85, 0.96]**	**[0.92, 0.98]**	

*Note:* 95% bias‐corrected and accelerated (BCa, *N* = 1000) bootstrapped confidence intervals for the correlations between illusions and IAF (IAF), using all parietal–occipital electrodes. The confidence intervals for the electrode subset O1, Oz and O2 are presented in Table S3. Confidence intervals that exclude zero are shown in **bold red font**. For all the correlations of the illusions with the IAF measures, the confidence intervals include zero. The blue cells show the confidence intervals for correlations not controlling for age. The red cells show the confidence intervals for correlations controlling for age.

It has been noted that IAF varies with age and is slower in older adults (Grandy et al. 2013). Thus, we wondered whether the absence of statistically significant correlations between the illusions and IAF was a consequence of not controlling for age effects with IAF. Therefore, we calculated a partial correlation to control for the effects of participants' age (Figure S12; see Table S2 for *p*‐values). The partial correlation replicated the correlations above, with a statistically significant correlation between the three measures of IAF, but no significant correlations between any of the illusions or between the illusions and IAF measures. Inspection of the confidence intervals again found that all confidence intervals included zero. Again, confirming that in the present study we have not found evidence for an effect.

Previous studies that have found a correlation between visual perception and IAF often only analyse data from a specific subset of electrodes (e.g., O1, Oz and O2; Cecere et al. 2015; Howard et al. 2017). In the present study, we analysed the data from 19 electrodes over the occipital and parietal cortex, making it possible that we could have been tapping into a mixture of oscillatory sources. Therefore, we repeated the correlation analysis using only the occipital electrodes typically used in past research. Focusing the data on only three electrodes resulted in more missing data, as participants required an IAF estimate for two of the three channels of interest in order to calculate the PAF and COG. As a result, PAF (*M* = 10.36, SD = 0.83, range = 8.5–12.33) could be estimated for 58 participants, and COG for 60 participants (*M* = 10.16, SD = 0.86, range = 8.23–12.2). Of the five eyes‐closed resting state trials, F_PAF was estimated for 60 participants (*M* = 10.32, SD = 0.83, range = 8.47–12.16).

The non‐age corrected and age‐corrected correlation matrices repeated with a subset of three occipital electrodes (Figure S13) replicated the patterns reported above, with only the measures of IAF significantly intercorrelating with one another (see Table S3 for confidence intervals). Overall, all four correlation analyses provide no evidence for a correlation between the three measures of IAF and any illusion magnitudes across eight different motion‐position illusions. This suggests that the magnitude of motion‐position illusions is not predicted by IAF.

### 3.3 Correlations Between Illusions

Regarding the relationships between the illusions themselves, after Bonferroni–Holm correction (for 28 pairwise comparisons), we did not replicate the pattern of statistical significance found by Cottier et al. (2023), who reported significant correlations between the Fröhlich and flash‐drag effect (0.37), between the twinkle‐goes and motion‐induced position shift (0.39), and between the twinkle‐goes and flash‐grab effect (0.38). Qualitatively, however, while not reaching significance after correcting for multiple comparisons, the pattern of correlations nevertheless appeared similar to those reported by Cottier et al. (2023), as described next.

**FIGURE 5:**
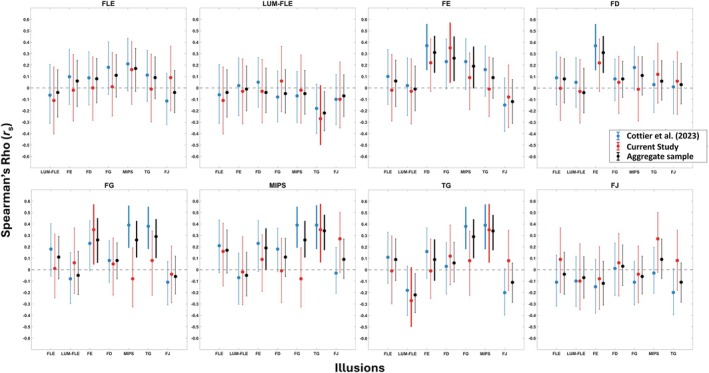
Bootstrapped confidence intervals for each illusion, for each study. *Note:* Error bars show the 95% bias‐corrected and accelerated (BCa) bootstrapped (*N* = 1000) confidence intervals. Blue colours show the correlations and confidence intervals for Cottier et al. (2023). The red colours show correlations and confidence intervals for the present study. The black colours show the confidence intervals with the aggregate sample. Confidence intervals that do not contain zero are represented by bold lines. Rgb values for the colours were obtained from the ColorBrewer website (Brewer et al., 2013).

To further explore the extent to which the correlation estimates were similar across the two studies, we plotted the 95% BCa confidence intervals for each study in Figure 5. These confidence intervals show that there is a great deal of similarity in the correlation estimates between the present study and Cottier et al. (2023). For almost all pairs of illusions, there is ample overlap between the confidence intervals in the two studies. The only exception is for FG and MIPS, where the previous study yielded strong evidence for a correlation, but the present study did not. However, the lack of statistical significance after Bonferroni‐Holm correction for the correlations in the present study suggests that although some correlations may be present, large sample sizes would be needed to reliably detect them.

As we had new participants that completed the illusions from Cottier et al. (2023), we were interested in obtaining an updated version of the intercorrelation matrix presented by Cottier et al. (2023, fig. 4). To this end, we created an aggregate dataset of 149 participants, comprising the 43 unique participants from the present study and 106 participants from Cottier et al. (2023). The correlation analyses were repeated with this aggregate sample, and the updated correlation matrix is presented in Figure S14. The *p*‐values for the updated correlation matrix are presented in Table S4. Overall, this auxiliary analysis replicated the key findings of Cottier et al. (2023). This is discussed in more detail in the Supporting Information. In Figure 5, we present the confidence intervals for the aggregate sample correlations. Ultimately, there is less variance in the confidence intervals for the aggregate correlations, indicating more precise correlation estimates. Overall, the conclusions to be drawn from this analysis are the same as those in Cottier et al. (2023) in that we observe evidence of weak to no correlation between the different illusions.

## 4 Discussion

Examination of individual differences can allow us to better understand the mechanistic structure of visual perception. Previous work suggested a relationship between IAF and perceptual phenomena, leading to the suggestion that IAF may index the temporal resolution of perception. While some MPIs are thought to be related to temporal resolution (e.g. Schneider, 2018; Linares et al. 2009), here, we found no statistically significant correlations between resting‐state IAF and eight different MPIs.

### 4.1 Absence of Correlations Between IAF and MPIs

The absence of correlations seems unlikely to be due to insufficient statistical power. Samaha and Romei (2024) found that the population correlation coefficient for the correlation between IAF and behavioural measures was typically between *r* = 0.39 and 0.53. Our sample size of 61 participants had 90% statistical power to detect relationships with a correlation coefficient above 0.37. Thus, our study was sufficiently powered to detect effects of the magnitude typically observed between IAF and behavioural measures (Samaha and Romei 2024).

If a relationship between IAF and MPIs does exist, then its magnitude is likely to be much smaller than the relationship previously observed between IAF and other behavioural measures. Weak correlations could have been hidden by participants' internal noise (Deodato and Melcher 2024). For example, Deodato and Melcher (2024) found that they could only replicate the correlation between IAF and the two‐flash fusion task reported by Samaha and Postle (2015) after using the slope of the psychometric function to control for participants' internal noise. This suggests that participants' internal noise can make it difficult to find a link between IAF and behavioural measures. The present study did not estimate participants' psychometric functions and is unable to implement this approach. As such, in the present study, it remains possible that participants' internal noise may have masked weak correlations between IAF and MPIs. Future research could adopt Deodato and Melcher's (2024) approach to minimise the effect noise may have on the correlation estimates.

Some of the tasks previously found to correlate with IAF do not contain any motion, as they are cross‐modal audio‐visual tasks or tasks designed to estimate the thresholds of perception. Of those that did involve motion, possibly important differences remain (Howard et al. 2017; Minami and Amano 2017; Ronconi et al. 2023; Shen et al. 2019; Zhang et al. 2019). For example, Ronconi et al. (2023) used the stream‐bounce illusion, which is an audio‐visual paradigm. The apparent motion Ternus display used by Shen et al. (2019) is a bistable stimulus. Zhang et al. (2019) used a bistable colour‐motion feature binding task. It is possible that some aspect of these paradigms does correlate with IAF but is absent from MPIs. Additionally, in the case of Shen et al. (2019), they looked at pre‐stimulus alpha before the task, whereas the present task looked at resting‐state alpha, which may have a weaker correlation with behavioural tasks. Overall, it seems that although IAF is implicated in various aspects of visual perception, including motion tasks, resting‐state IAF plays small to no role in MPIs. It is important to note that our results are constrained strictly to resting‐state IAF. It is possible that task‐based IAF is involved with the perception of MPIs. This is a question for future research to address.

### 4.2 Absence of Correlations Between Illusions

In our sample of 61 participants, after correcting for multiple comparisons, we did not replicate the statistically significant correlations reported by Cottier et al. (2023). However, as shown in Figure 5, the correlation estimates were nevertheless similar across studies. A natural explanation for the absence of statistically significant correlations in the present study is the smaller sample size in the present study (61 vs. 106 in Cottier et al. 2023). However, statistically significant effects in Cottier et al. (2023) had correlation coefficients of 0.37 or higher and based on our sample size the current study had 90% power to detect effects of this size (albeit with an uncorrected *p*‐value of 0.05). However, participants completed fewer trials per illusion, viewing these illusions once instead of twice, as in Cottier et al. (2023), which increased the variability and effectively further reduced the statistical power. Therefore, it seems possible that the correlations between illusions might be truly smaller than reported in Cottier et al. (2023). This is supported by our confidence interval and correlation estimates, which show the estimated correlation with the aggregate sample was smaller than reported in Cottier et al. (2023). However, as pointed out by a helpful reviewer, the observed absence of correlations simply could be due to noise, particularly as our estimates of our illusory effect are noisier in the present study than in the previous study by Cottier et al. (2023).

### 4.3 Discrete Sampling at Alpha Is Unlikely to Account for MPIs

Based on the longstanding perceptual moment hypothesis (Stroud 1967), Schneider (2018) proposed that discrete sampling could explain the FLE, FE and other MIPS effects. Under the discrete sampling hypothesis for visual processing, the temporal resolution which IAF may index (Morrow and Samaha 2022) would correspond to the duration of the visual system's sampling window, and thus IAF should correlate with illusion magnitude (Morrow and Samaha 2022). Our finding of no evidence for correlations between IAF and MPIs challenges the discrete sampling account of these illusions and suggests it might not be an underlying cause of these effects. This interpretation is corroborated by our observation that the FLE, FE and FJ did not correlate with one another, as Cottier et al. (2023) and Morrow and Samaha (2022) found. Under discrete sampling, these illusions should correlate. Our results therefore suggest that discrete sampling alpha is not involved in these illusions. However, we are not able to rule out the possibility that these illusions are driven by discrete sampling at different oscillation frequencies (Morrow and Samaha 2022), or by trial‐level sampling processes which are independent from resting state mechanisms (see below). Furthermore, we cannot rule out the possibility of there being very small correlations between IAF and MPIs that this study was not sufficiently powered to detect.

Previous research has linked ongoing trial‐level alpha dynamics (e.g., phase) to FLE magnitude (Chakravarthi and VanRullen 2012; Chota and VanRullen 2019). In the present study, we found no evidence for a link between trait‐based components of alpha and the FLE. This difference in results may be due to the fact that the present study looked at resting state alpha dynamics recorded in a separate session to when participants completed the illusions, while previous studies have recorded EEG as participants complete the illusions. Thus, there could be some aspect of alpha (e.g., peristimulus phase) that is related to illusion magnitude, which the present study was not designed to detect. Given that peristimulus alpha dynamics (like phase) have been related to illusory perception (Cecere et al. 2015; Chakravarthi and VanRullen 2012; Lange et al. 2014; Samaha and Postle 2015) and that the position of moving objects can be decoded from ongoing trial‐level alpha power (Turner et al. 2023), future research should explore how single‐trial oscillatory dynamics mediate the perception of MPIs. Additionally, this research will help us infer the extent to which attentional processes are related to the perception of these illusions. There is a large body of literature showing peristimulus alpha dynamics appear to represent neural correlates of attentional inhibition (see Bonnefond and Jensen 2025).

By focusing exclusively on resting‐state IAF, this paper is constrained in its ability to test discrete sampling. To emphasise our point, this paper simply shows that resting‐state IAF, and discrete sampling as a trait‐based process indexed by alpha is unlikely to be the cause of these illusions. However, given the substantial body of literature showing that task‐based peristimulus alpha modulates and is related to visual perception (Bonnefond and Jensen 2025; VanRullen 2016), we might expect task‐based alpha to correlate with illusory perception (as previously shown with the FLE by Chakravarthi and VanRullen 2012 and Chota and VanRullen 2019). Our results instead are consistent with the notion that if perception is due to discrete sampling, it is not due to discrete sampling at a single perceptual rhythm of alpha (VanRullen 2016). It is possible that perception is a consequence of discrete sampling at multiple different temporal resolutions, resolutions which may have longer windows of integration (Herzog et al. 2020; VanRullen 2016). Therefore, it remains possible that these illusions are modulated by discrete sampling at oscillatory frequencies other than alpha (Morrow and Samaha 2022).

## 5 Conclusion

Using an individual differences approach, the present study explored whether resting state IAF could predict the magnitude of eight MPIs. Correlation analyses found no evidence of an association between IAF and any of the illusions, suggesting that alpha‐linked discrete sampling of visual information is not responsible for any of these effects. After correcting for multiple comparisons, we did not replicate the statistically significant effects reported in Cottier et al. (2023). However, confidence intervals revealed the correlation estimates were nevertheless similar across studies. An auxiliary analysis of aggregate data across these studies yielded updated and more precise estimates of inter‐illusion correlations—overall showing evidence of weak to no association between these effects. Future research may explore how ongoing trial‐to‐trial oscillatory dynamics relate to MPIs. This would help to further characterise the extent to which neural oscillations influence motion and position perception.

## Author Contributions

**Timothy Cottier:** conceptualization, writing – original draft, review and editing, formal analysis and investigation. **William Turner:** conceptualization, supervision, writing – review and editing. **Violet J. Chae:** investigation, writing – review and editing. **Alex O. Holcombe:** writing – review and editing. **Hinze Hogendoorn:** conceptualization, supervision, funding acquisition, writing – review and editing.

## Ethics Statement

Ethics approval was granted by the University of Melbourne human research ethics committee (Illusion ID: 2022‐12816‐29275‐8, EEG ID: 2022‐12985‐29276‐6).

## Conflicts of Interest

The authors declare no conflicts of interest.

## Peer Review

The peer review history for this article is available at https://www.webofscience.com/api/gateway/wos/peer‐review/10.1111/ejn.70250.

## Supporting information




**Figure S1:** Histograms displaying the distribution for each illusion and IAF. **Table S1:** Kolmogorov‐Smirnov tests for each illusion and IAF (IAF). **Figure S2:** Scatterplots showing participants scores for the Flash lag effect, the other illusions, and peak alpha frequency. **Figure S3:** Scatterplots showing participants scores for the Luminance flash lag effect, the other illusions, and peak alpha frequency. **Figure S4:** Scatterplots showing participants scores for the Fröhlich effect, the other illusions, and peak alpha frequency. **Figure S5:** Scatterplots showing participants scores for the Flash‐drag effect, the other illusions, and peak alpha frequency. **Figure S6:** Scatterplots showing participants scores for the Flash‐grab effect, the other illusions, and peak alpha frequency. **Figure S7:** Scatterplots showing participants scores for the Motion‐induced position shift, the other illusions, and peak alpha frequency. **Figure S8:** Scatterplots showing participants scores for the Twinkle‐goes effect, the other illusion, and peak alpha frequency. **Figure S9:** Scatterplots showing participants scores for the Flash‐jump effect, the other illusions, and peak alpha frequency. **Figure S10:** Scatterplots between participants Centre of Gravity and the other illusions. **Figure S11:** Scatterplots between participants FOOOF Peak Alpha Frequency and the other illusions. **Figure S12:** Correlations between each illusions and IAF measure, controlling for age. **Table S2:** P values for each correlation analysis between the illusions and IAF. **Figure S13:** Correlations between IAF and the illusions, with IAF calculated with the data from a subset of electrodes (Oz, O1, and O2). **Table S3:** Bootstrapped confidence intervals for the correlations between illusions and IAF (IAF), when IAF is calculated only using the data from electrodes O1, Oz, and O2. **Figure S14:** Correlations between the motion‐position illusions using the aggregate sample. **Table S4:** P‐values for the correlation analysis between the illusions, using the aggregate sample
.

## Data Availability

Upon publication, the experiment code, analysis code, raw EEG data and processed behavioural data will be made available at this link: https://osf.io/jxeu3/.
