# Supplementary material for: No Evidence That Resting‐State Individual Alpha Frequency Represents a Mechanism Underlying Motion‐Position Illusions
Source: Eur J Neurosci. 2025 Nov 2;62(9):e70250. doi: 10.1111/ejn.70250 (PMC12580576; doi:10.1111/ejn.70250)
Supplement: Supplementary file 1 — Figure S1: Histograms displaying the distribution for each illusion and IAF. Table S1: Kolmogorov‐Smirnov tests for each illusion and IAF (IAF). Figure S2: Scatterplots showing participants scores for the Flash lag effect, the other illusions, and peak alpha frequency. Figure S3: Scatterplots showing participants scores for the Luminance flash lag effect, the other illusions, and peak alpha frequency. Figure S4: Scatterplots showing participants scores for the Fröhlich effect, the other illusions, and peak alpha frequency. Figure S5: Scatterplots showing participants scores for the Flash‐drag effect, the other illusions, and peak alpha frequency. Figure S6: Scatterplots showing participants scores for the Flash‐grab effect, the other illusions, and peak alpha frequency. Figure S7: Scatterplots showing participants scores for the Motion‐induced position shift, the other illusions, and peak alpha frequency. Figure S8: Scatterplots showing participants scores for the Twinkle‐goes effect, the other illusion, and peak alpha frequency. Figure S9: Scatterplots showing participants scores for the Flash‐jump effect, the other illusions, and peak alpha frequency. Figure S10: Scatterplots between participants Centre of Gravity and the other illusions. Figure S11: Scatterplots between participants FOOOF Peak Alpha Frequency and the other illusions. Figure S12: Correlations between each illusions and IAF measure, controlling for age. Table S2: P values for each correlation analysis between the illusions and IAF. Figure S13: Correlations between IAF and the illusions, with IAF calculated with the data from a subset of electrodes (Oz, O1, and O2). Table S3: Bootstrapped confidence intervals for the correlations between illusions and IAF (IAF), when IAF is calculated only using the data from electrodes O1, Oz, and O2. Figure S14: Correlations between the motion‐position illusions using the aggregate sample. Table S4: P‐values for the correlation analysis between the illusions, usin [file EJN-62-0-s001.docx]

# 7.0 Supplementary materials

***Supplementary materials Figure 1.*** Histograms displaying the distribution for each illusion and IAF.

**
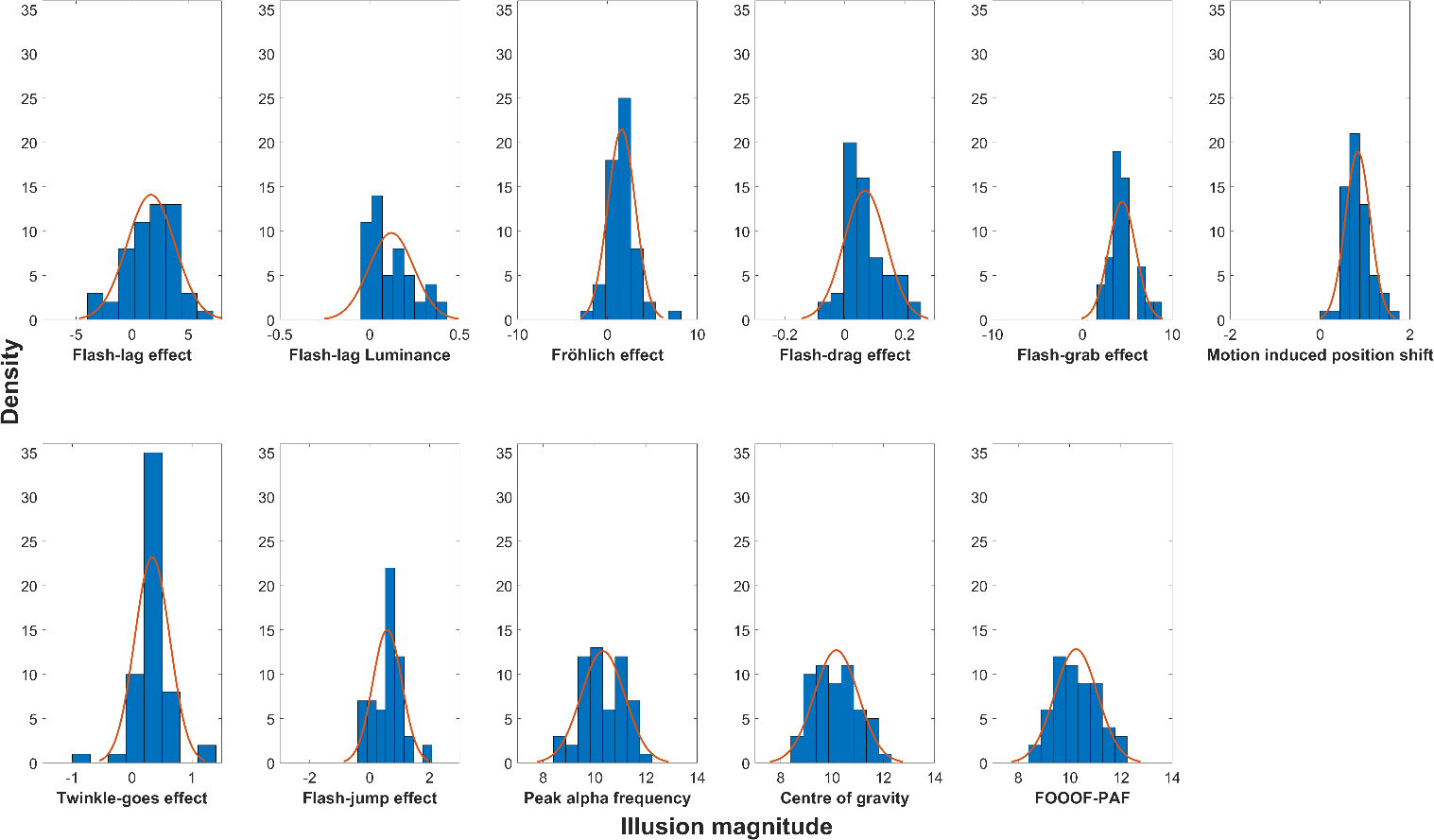
**

***Note.*** Distributions were fit using the default parameters of MATLAB’s *“HistFit”* function.

***Supplementary materials Table 1*.** Kolmogorov-Smirnov tests for each illusion and IAF (IAF).

| **Illusions** | **Kolmogorov-Smirnov Statistic (*df*)** |
| --- | --- |
| Flash-lag effect (FLE) | *D*(53) = 0.52 |
| Flash-lag luminance effect (LUM-FLE) | *D*(50) = 0.48 |
| Fröhlich effect (FE) | *D*(58) = 0.55 |
| Flash-drag effect (FD) | *D*(60) = 0.48 |
| Flash-grab effect (FG) | *D*(55) = 0.99 |
| Motion induced position shift (MIPS) | *D*(59) = 0.66 |
| Twinkle-goes (TG) | *D*(56) = 0.46 |
| Flash-jump (FJ) | *D*(58) = 0.42 |
| **Resting state EEG** |  |
| Peak Alpha Frequency (PAF) | *D*(60) = 1 |
| Centre of Gravity (COG) | *D*(60) = 1 |
| FOOOF PAF (F_PAF) | *D*(60) = 1 |

***Note.*** All Kolmogorov-Smirnov tests were significant (*p* < 0.001), suggesting the distributions were significantly different from a normal distribution.


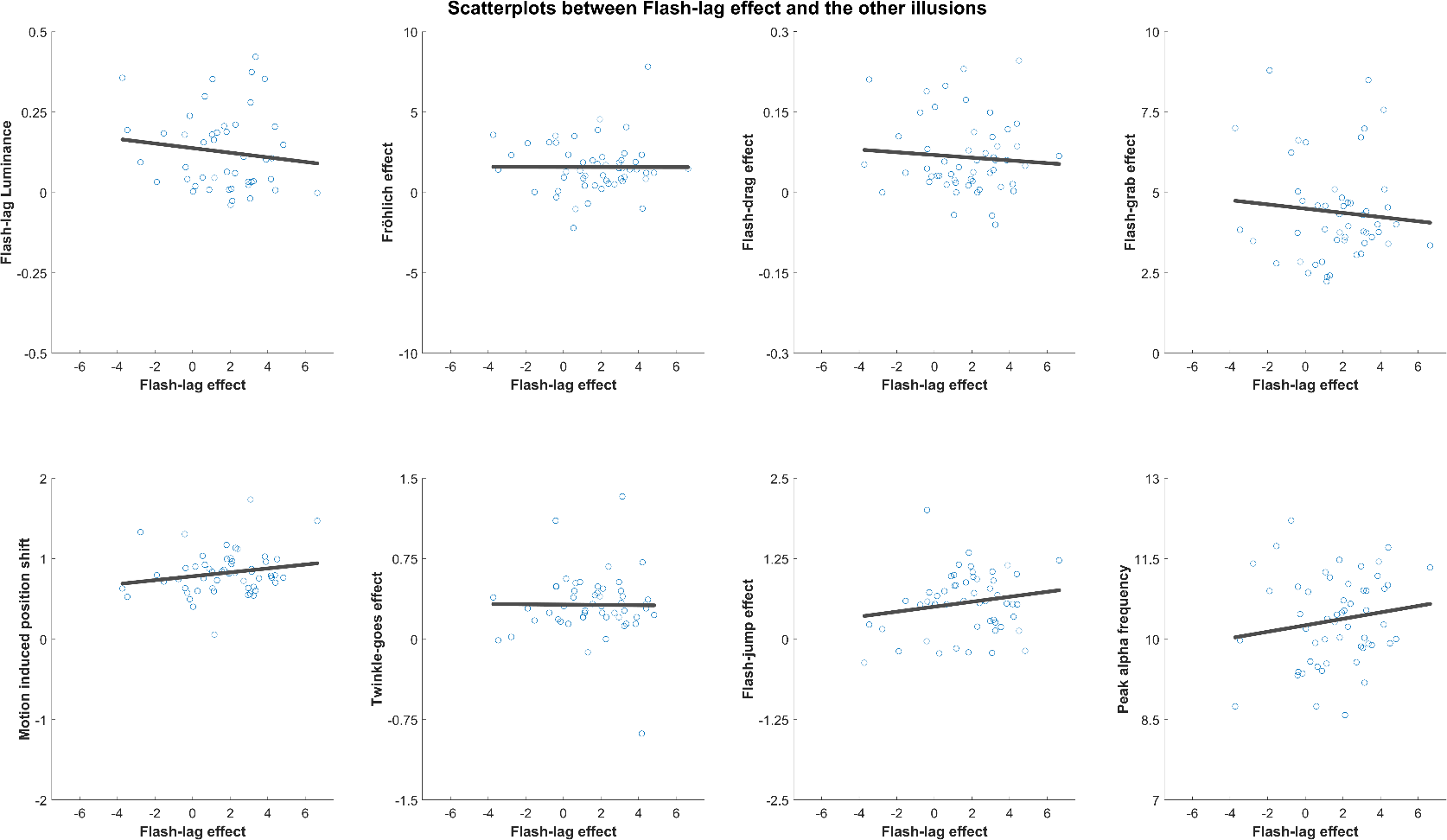
***Supplementary materials Figure 2.*** Scatterplots showing participants scores for the Flash lag effect, the other illusions, and peak alpha frequency.


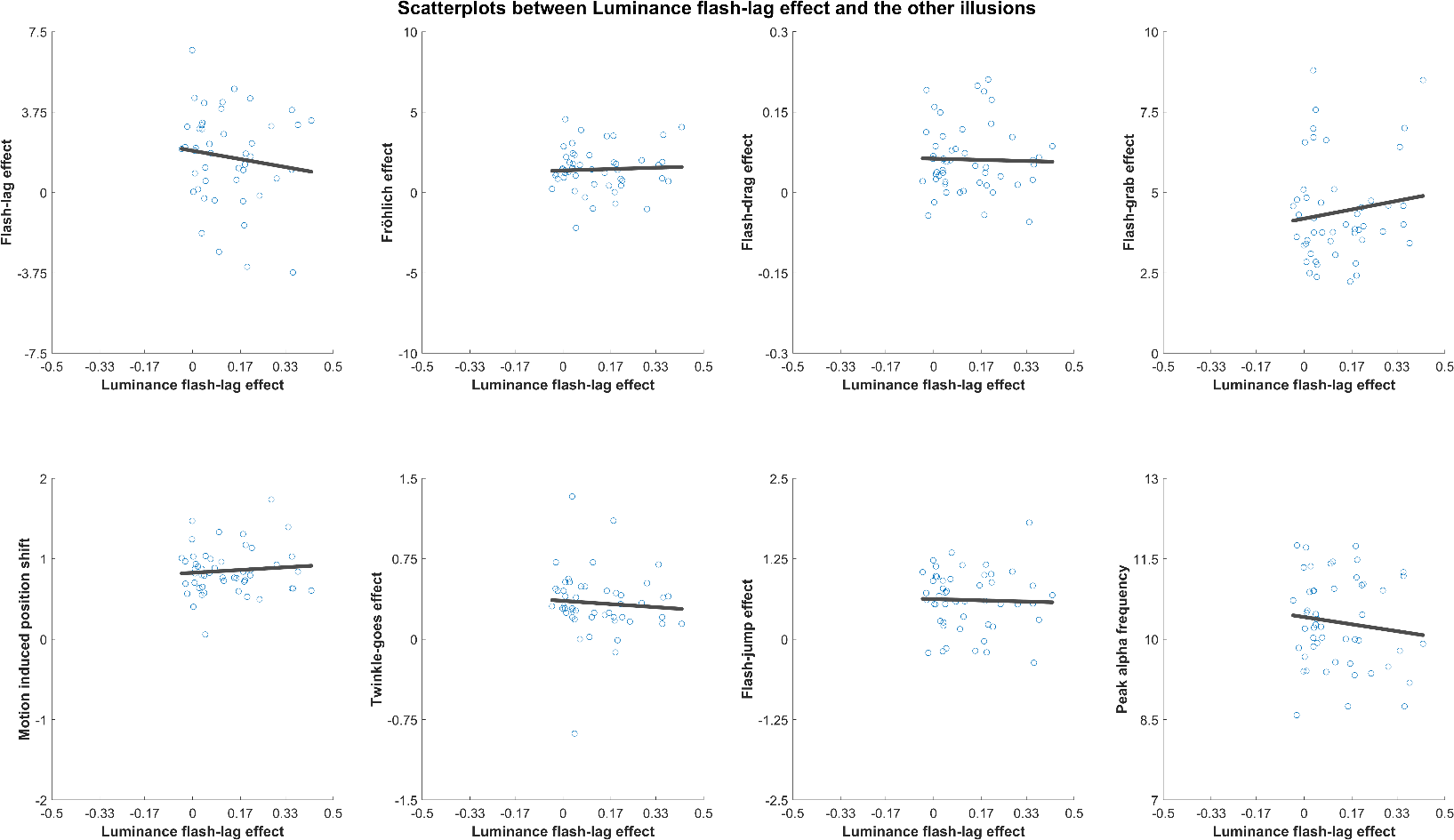
***Supplementary materials Figure 3.*** Scatterplots showing participants scores for the Luminance flash lag effect, the other illusions, and peak alpha frequency.


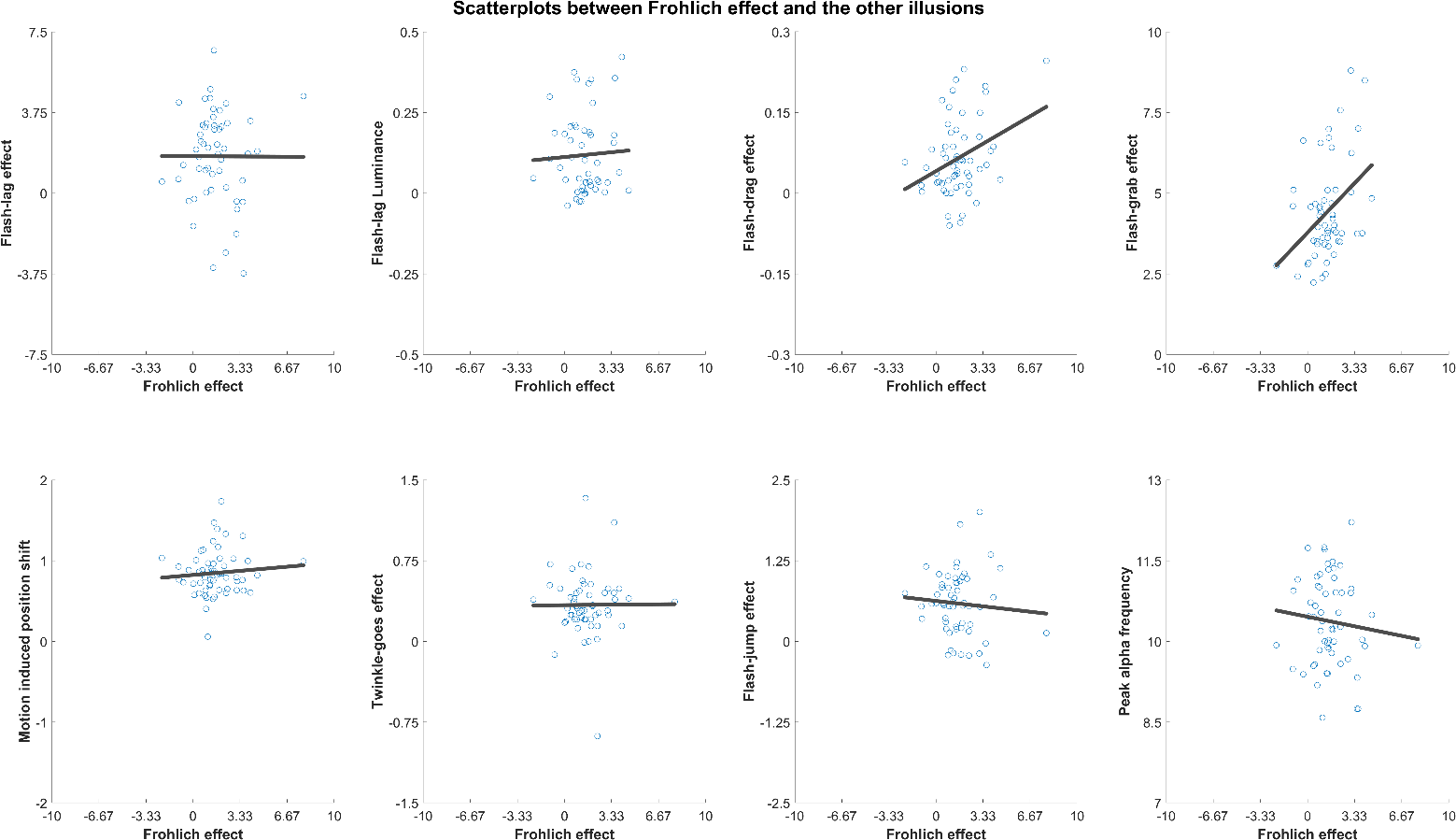
 ***Supplementary materials Figure 4.*** Scatterplots showing participants scores for the Fröhlich effect, the other illusions, and peak alpha frequency.


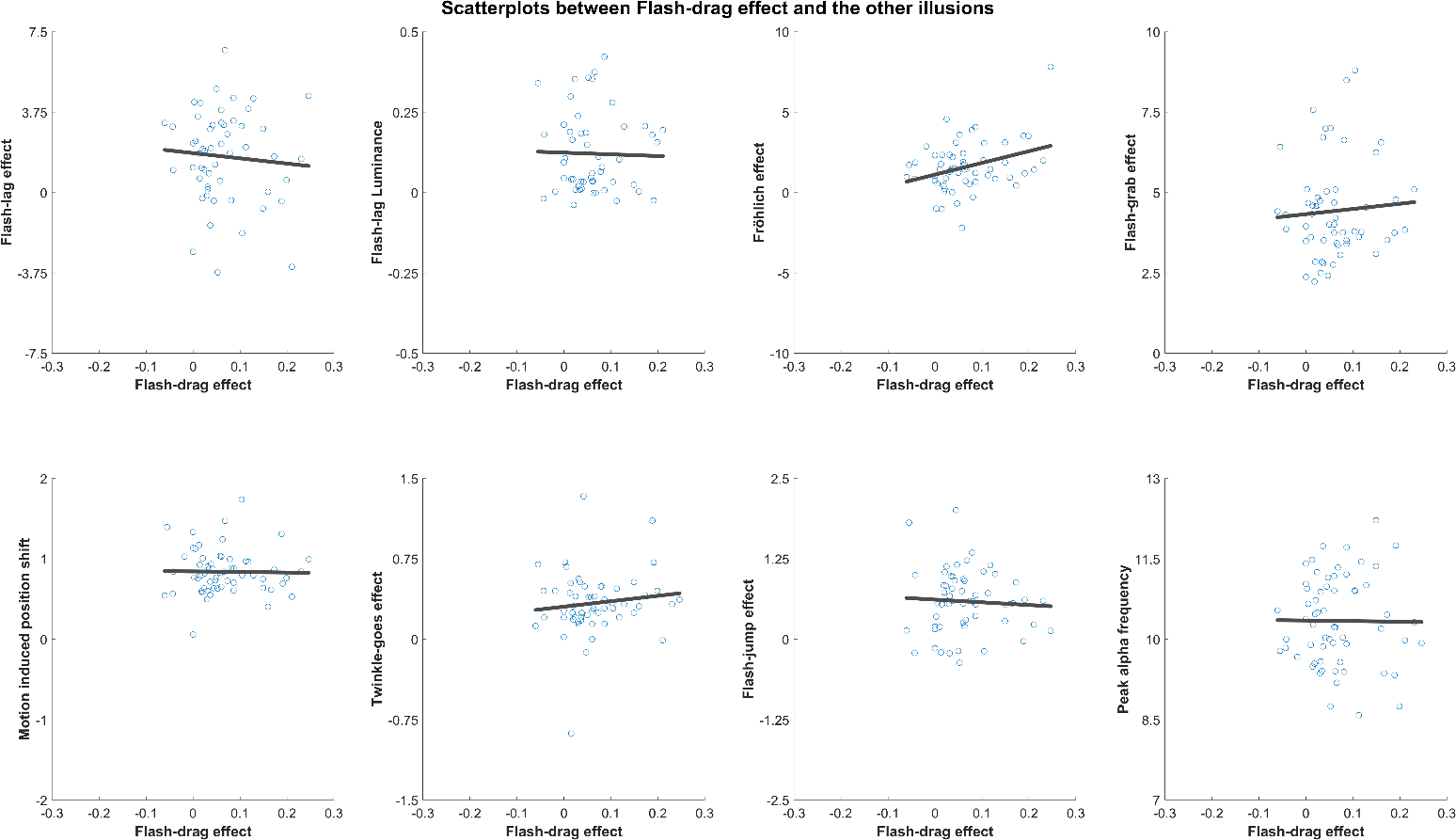
 ***Supplementary materials Figure 5.*** Scatterplots showing participants scores for the Flash-drag effect, the other illusions, and peak alpha frequency.

***Supplementary materials Figure 6.*** Scatterplots showing participants scores for the
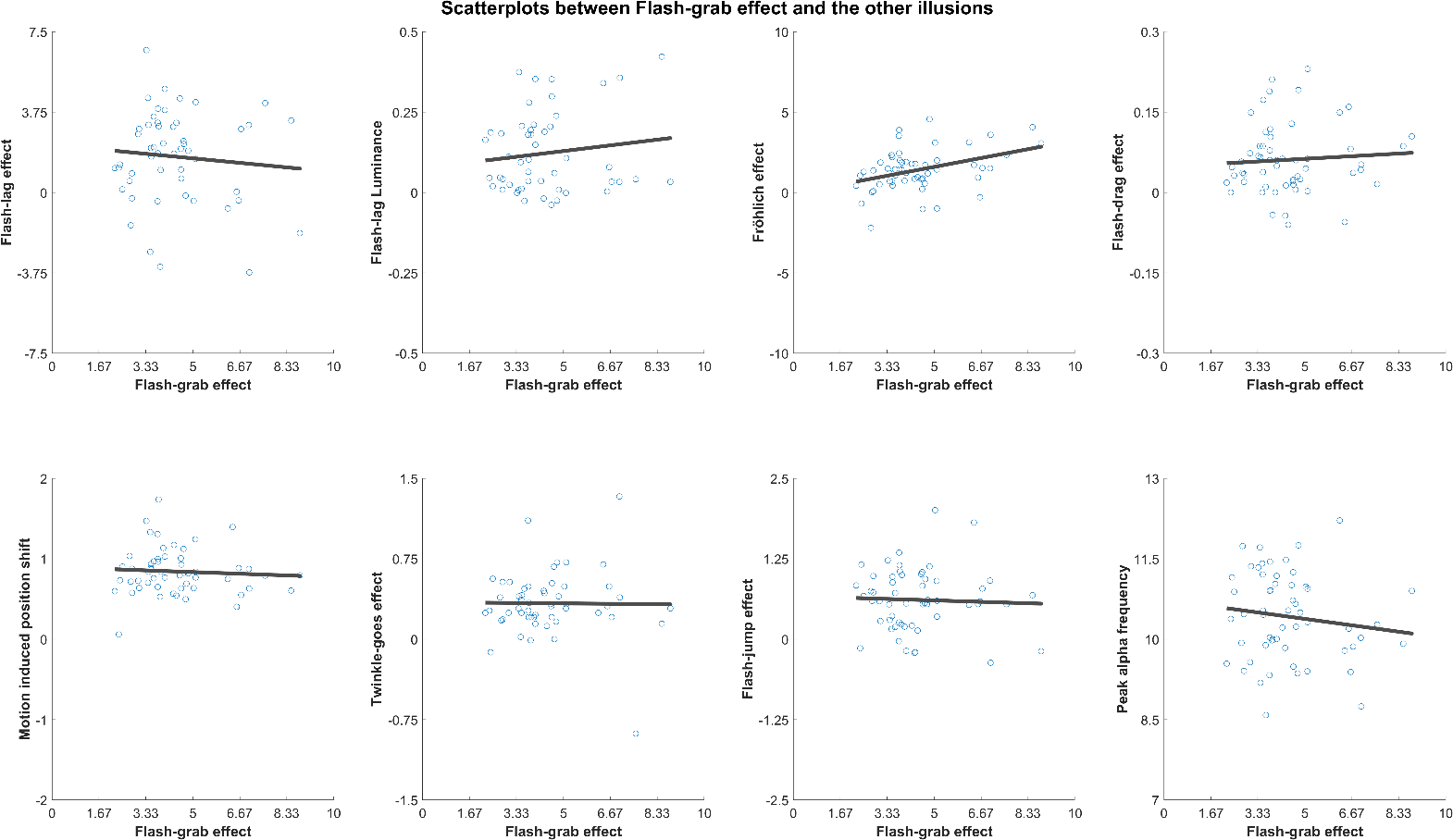
Flash-grab effect, the other illusions, and peak alpha frequency.


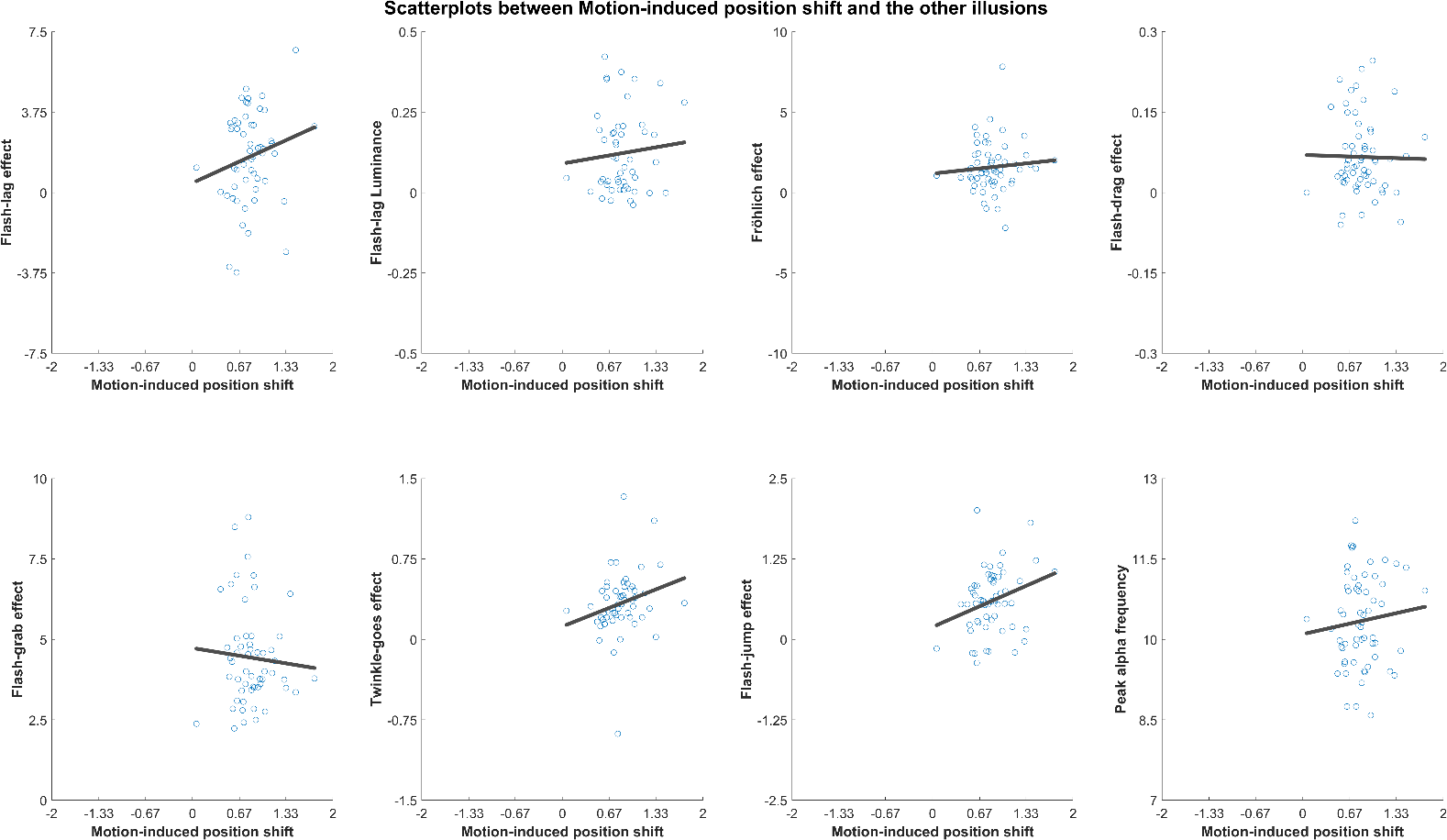
***Supplementary materials Figure 7.*** Scatterplots showing participants scores for the Motion-induced position shift, the other illusions, and peak alpha frequency.

***Supplementary materials Figure 8.*** Scatterplots showing participants scores for the Twinkle-goes effect, the other illusion, and peak alpha frequency.


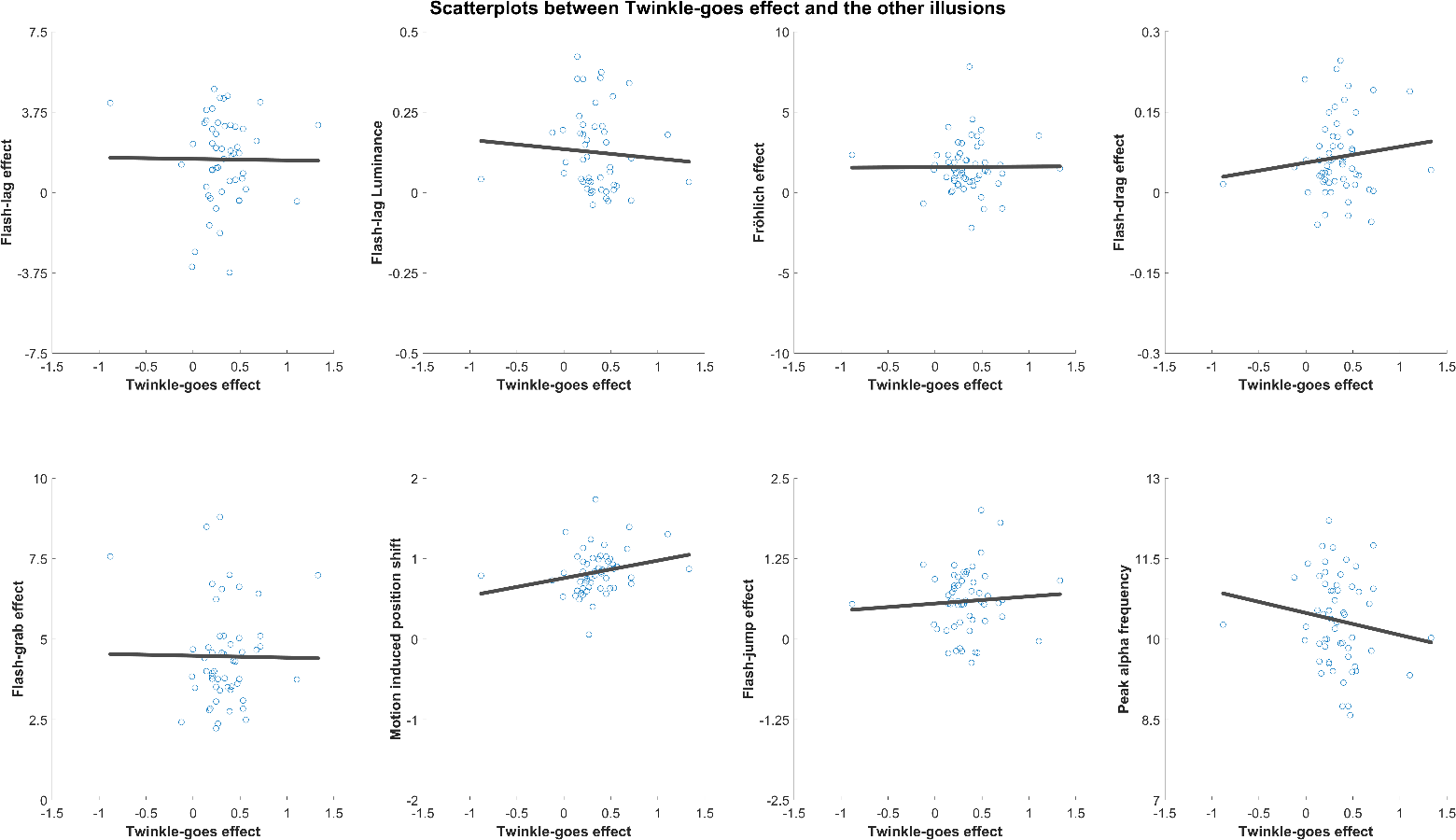


***Supplementary materials Figure 9.*** Scatterplots showing participants scores for the Flash-jump effect, the other illusions, and peak alpha frequency.


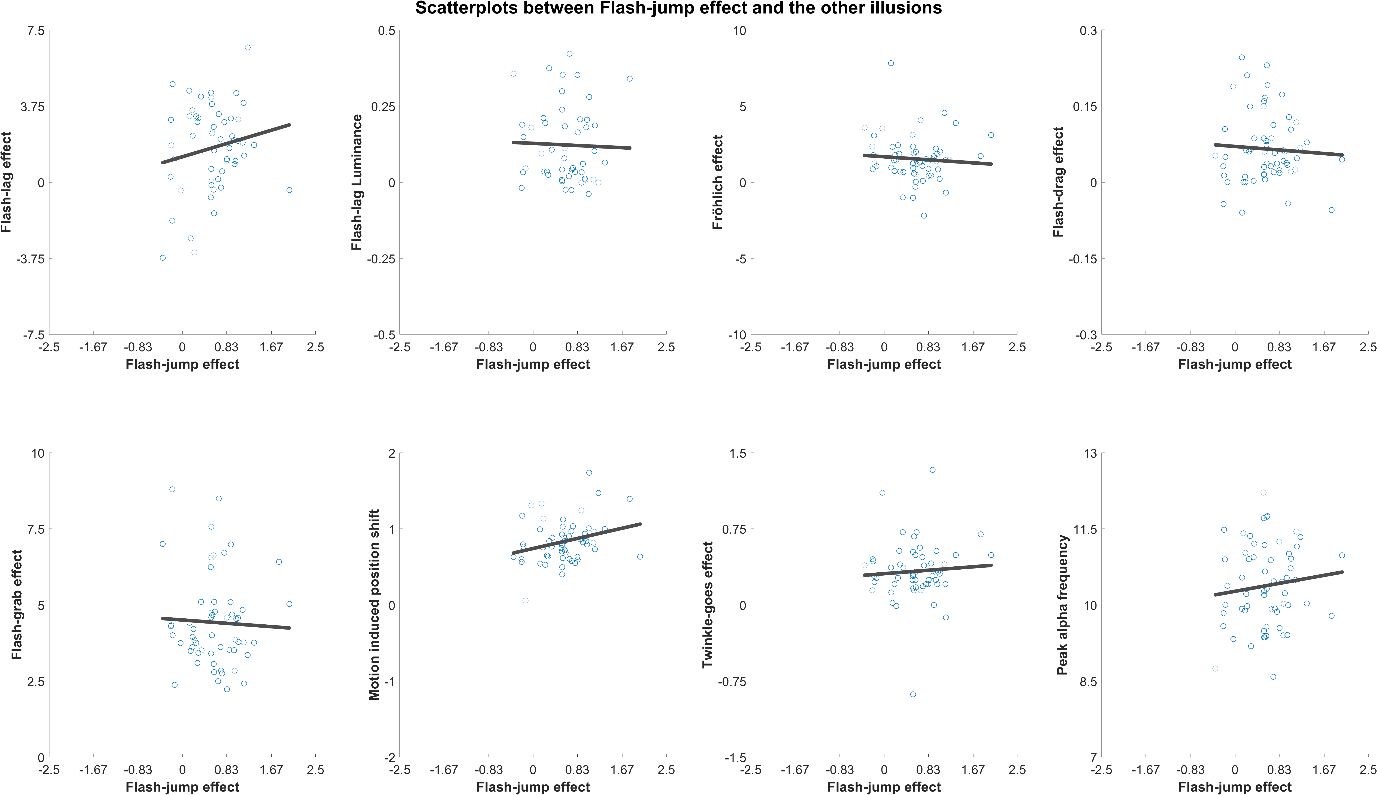


***Supplementary materials Figure 10 -*** Scatterplots between participants Centre of Gravity and the other illusions.


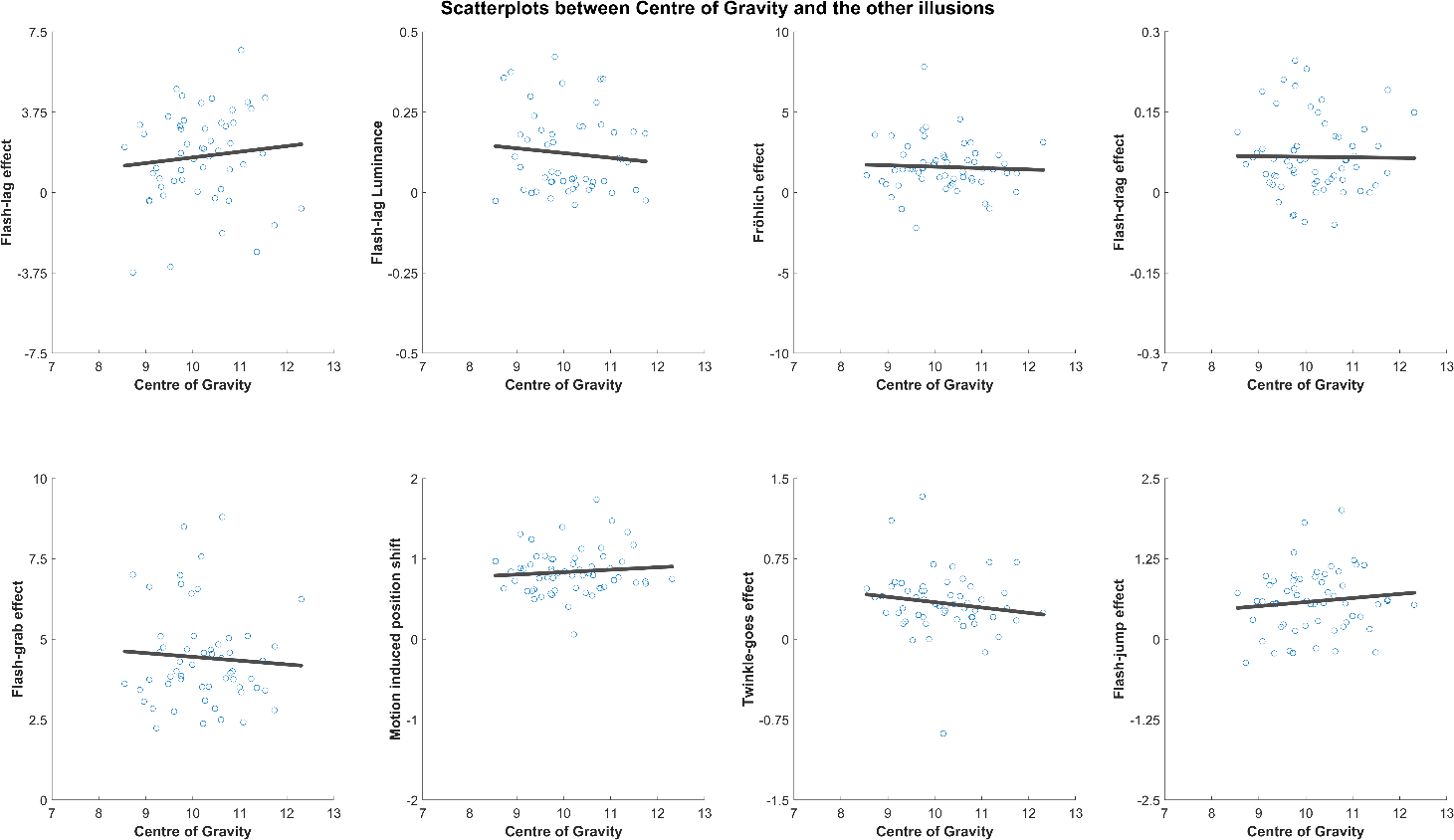


***Supplementary materials Figure 11*** - Scatterplots between participants FOOOF Peak Alpha Frequency and the other illusions.


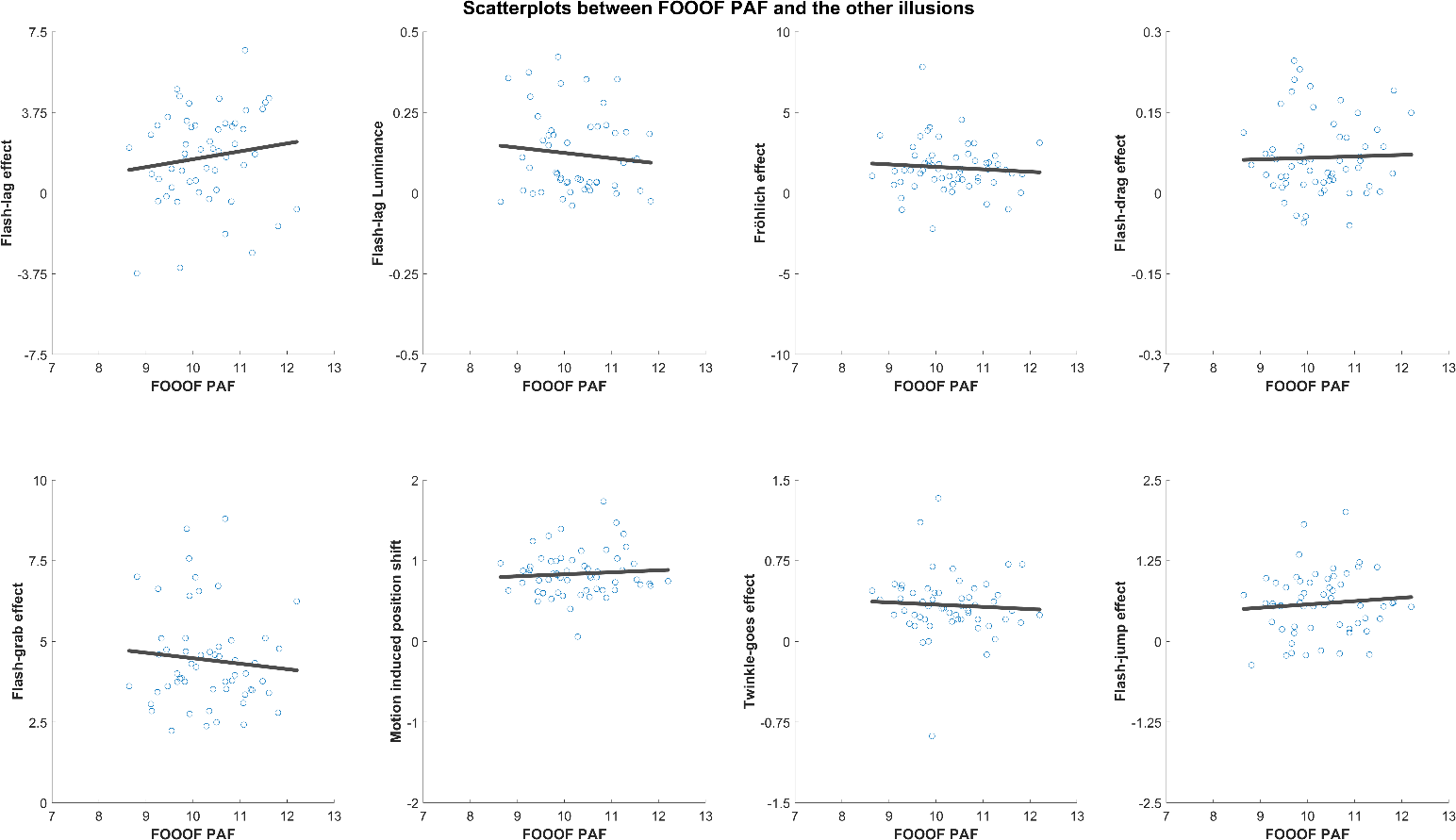


***Supplementary materials Figure 12****.* Correlations between each illusions and IAF measure, controlling for age.


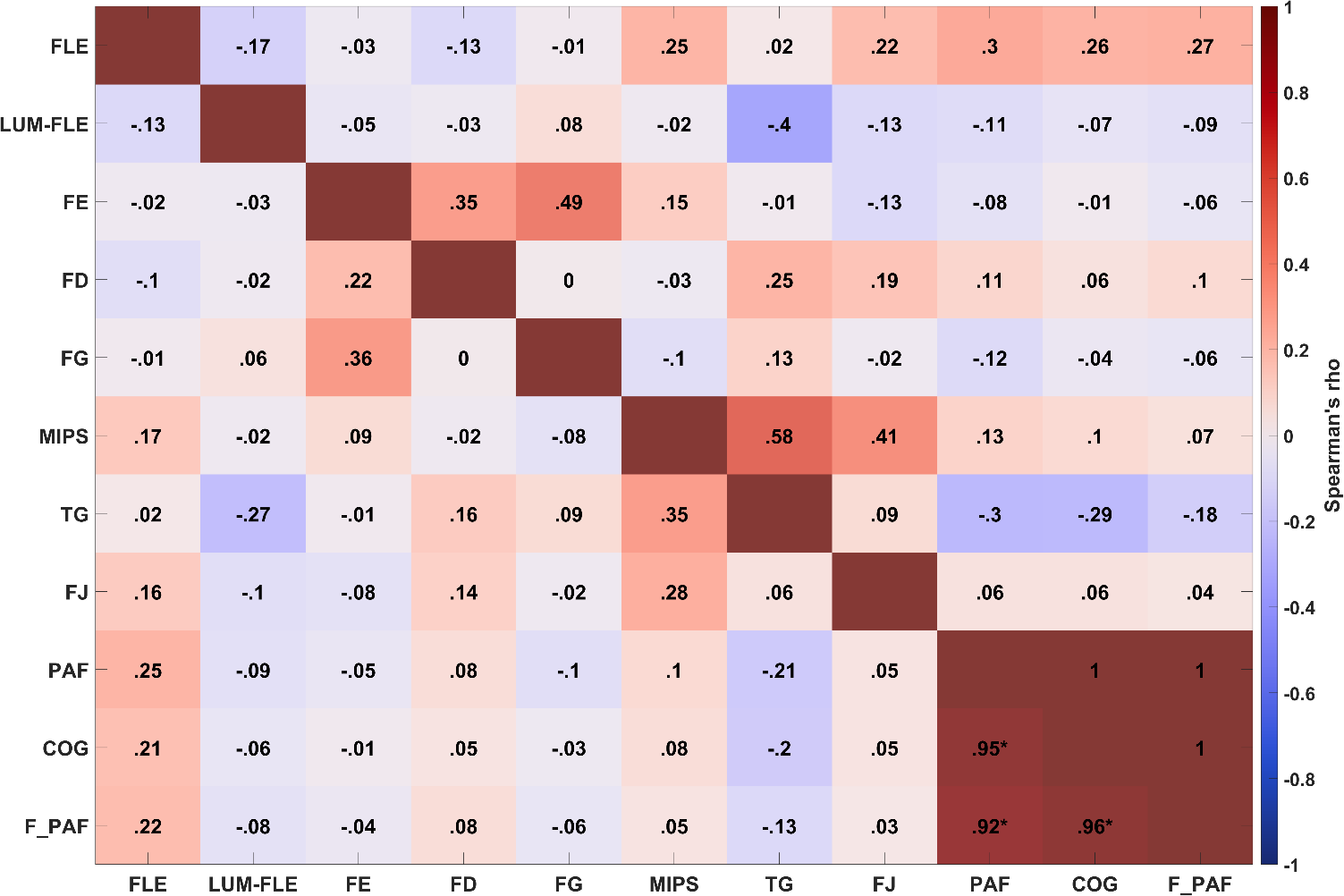


***Note.*** Measures of Individual Alpha Frequency (IAF) were calculated using EEG recorded over all parietal-occipital electrodes. Correlations were rounded to two decimal places. FLE = Flash-lag effect, LUM-FLE = luminance flash-lag effect, FE = Fröhlich effect, FD = flash-drag effect, FG = flash-grab effect, MIPS = motion-induced position shift, TG = twinkle-goes effect, FJ = flash-jump effect, PAF = peak alpha frequency, COG = centre of gravity, and F_PAF = peak alpha frequency with the aperiodic element removed. The disattenuated correlations are presented above the diagonal line, and the raw scores are presented below the diagonal. The p values for these correlations are presented in Supplementary materials table 2. Correlations not controlled for age correlations are presented in Figure 4.

| **All electrodes** | | | | | | | | | | | | |  | |
| --- | --- | --- | --- | --- | --- | --- | --- | --- | --- | --- | --- | --- | --- | --- |
| **Illusions** | FLE | LUM-FLE | | Fröhlich | FD | FG | MIPS | TG | FJ | PAF | COG | F_ PAF | |  |
| FLE |  | .381 | .905 | | .477 | .962 | .213 | .917 | .245 | .073 | .13 | .115 | |  |
| Lum-FLE | .458 |  | .832 | | .871 | .665 | .905 | .065 | .494 | .524 | .704 | .598 | |  |
| Fröhlich | .905 | .826 |  | | .094 | .008 | .489 | .963 | .548 | .691 | .948 | .753 | |  |
| FD | .988 | .994 | .098 | |  | .985 | .892 | .252 | .303 | .521 | .707 | .56 | |  |
| FG | .932 | .664 | .009 | | .736 |  | .575 | .526 | .912 | .457 | .821 | .684 | |  |
| MIPS | .248 | .904 | .484 | | .924 | .571 |  | .008 | .033 | .455 | .565 | .701 | |  |
| TG | .946 | .061 | .951 | | .388 | .588 | .007 |  | .665 | .119 | .132 | .348 | |  |
| FJ | .537 | .496 | .550 | | .663 | .771 | .038 | .549 |  | .711 | .726 | .811 | |  |
| PAF | .233 | .546 | .657 | | .962 | .332 | .485 | .202 | .445 |  | **<.001** | **<.001** | |  |
| COG | .309 | .722 | .909 | | .826 | .657 | .59 | .205 | .479 | **<.001** |  | **<.001** | |  |
| F_PAF | .257 | .61 | .722 | | .96 | .548 | .72 | .456 | .576 | **<.001** | **<.001** |  | |  |
| **Electrodes O1, Oz, O2** | | | | | | | | | | | | |  | |
| **Illusions** | FLE | LUM-FLE | Fröhlich | | FD | FG | MIPS | TG | FJ | PAF | COG | F_PAF | |  |
| FLE |  | .381 | .905 | | .477 | .962 | .213 | .917 | .245 | .182 | .107 | .161 | |  |
| Lum-FLE | .458 |  | .832 | | .871 | .665 | .905 | .065 | .494 | .65 | .571 | .46 | |  |
| Fröhlich | .905 | .826 |  | | .094 | .008 | .489 | .963 | .548 | .657 | .865 | .75 | |  |
| FD | .988 | .843 | .098 | |  | .985 | .892 | .252 | .303 | .492 | .936 | .673 | |  |
| FG | .932 | .664 | .009 | | .736 |  | .575 | .526 | .912 | .456 | .892 | .495 | |  |
| MIPS | .248 | .904 | .484 | | .924 | .571 |  | .008 | .033 | .314 | .651 | .842 | |  |
| TG | .946 | .061 | .951 | | .388 | .588 | .007 |  | .665 | .197 | .11 | .299 | |  |
| FJ | .537 | .496 | .550 | | .663 | .771 | .038 | .549 |  | .834 | .985 | .842 | |  |
| PAF | .442 | .75 | .763 | | .969 | .354 | .339 | .297 | .718 |  | **<.001** | **<.001** | |  |
| COG | .359 | .636 | .959 | | .473 | .715 | .661 | .177 | .604 | **<.001** |  | **<.001** | |  |
| F_ PAF | .412 | .441 | .691 | | .896 | .4 | .926 | .414 | .843 | **<.001** | **<.001** |  | |  |

***Supplementary materials Table 2.*** P values for each correlation analysis between the illusions and IAF. The blue cells show the p-values for non-age corrected correlations, and the red cells show the p-values for age-corrected correlations. For the parietal-occipital electrodes, the non-age corrected correlations can be found in figure 4, and the age corrected correlations are provided in Supplementary materials figure 12. The age corrected and non-age corrected correlations for electrode subset O1, Oz, and O2 are provided in Supplementary materials figure 13. Correlations statistically significant (*p* < .05) before Bonferroni-Holm correction, but not after Bonferroni correction are highlighted in red font. Correlations statistically significant after Bonferroni-holm corrected are **bolded.**

***Supplementary materials Figure 13.***


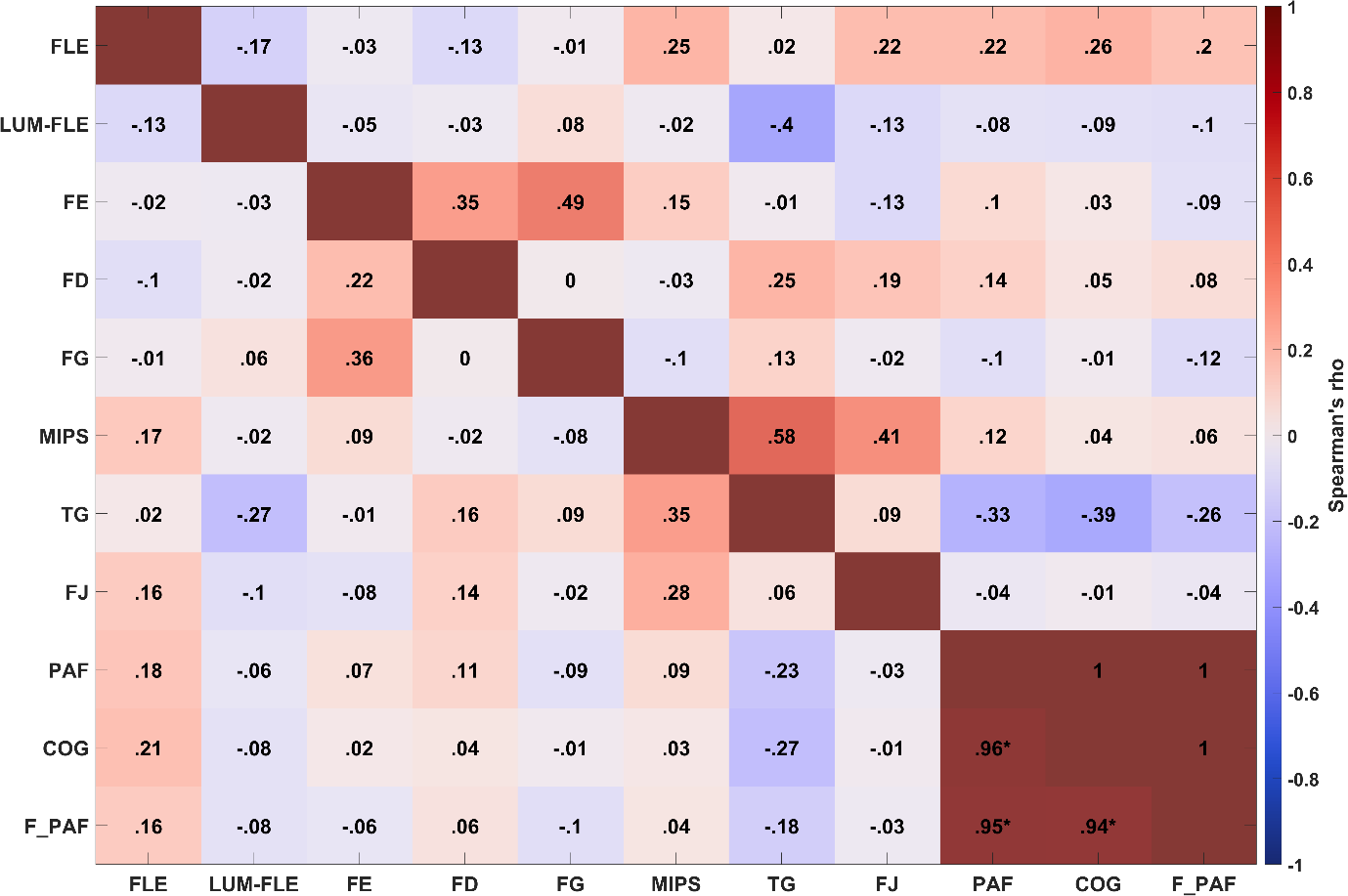

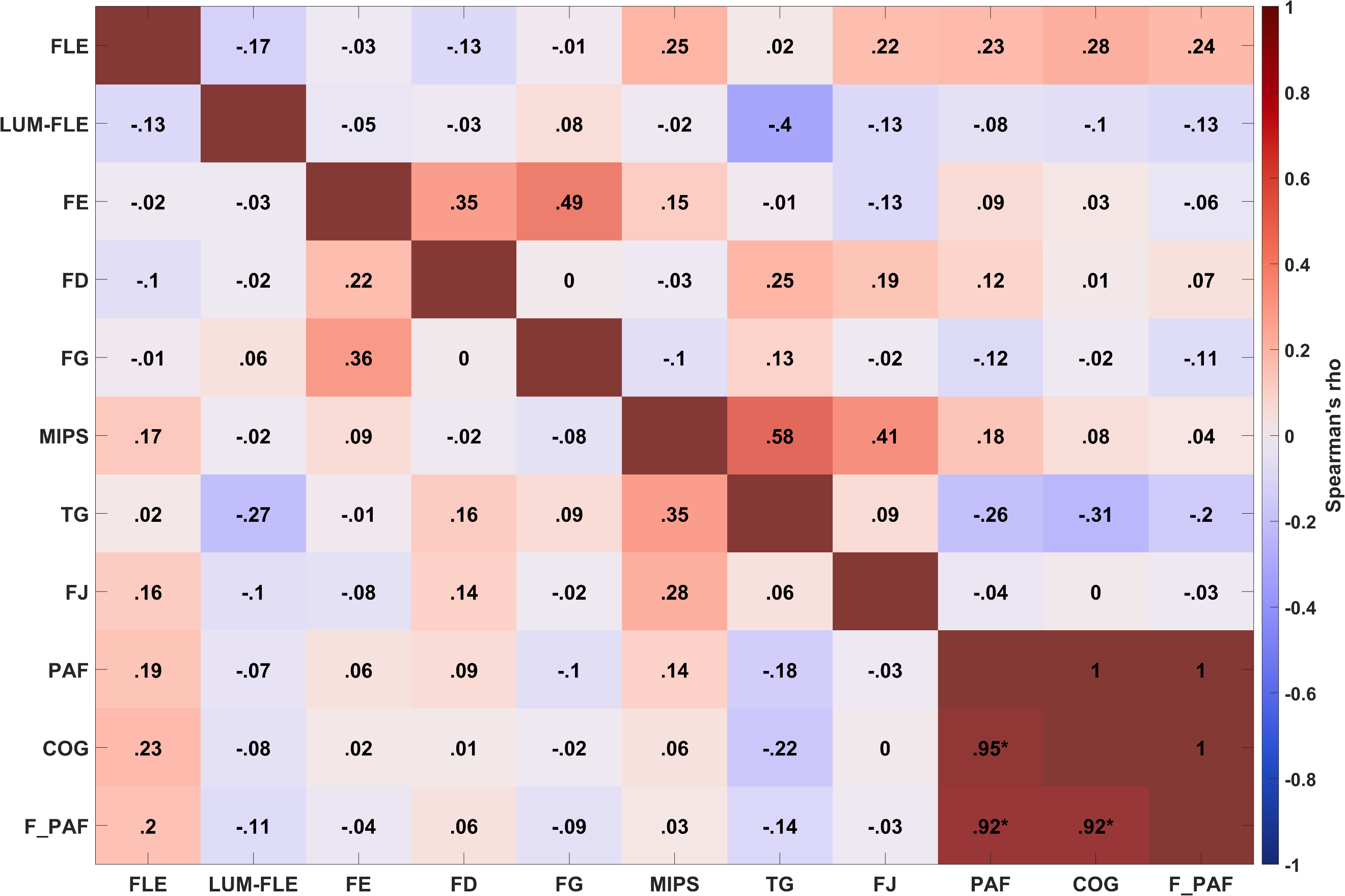

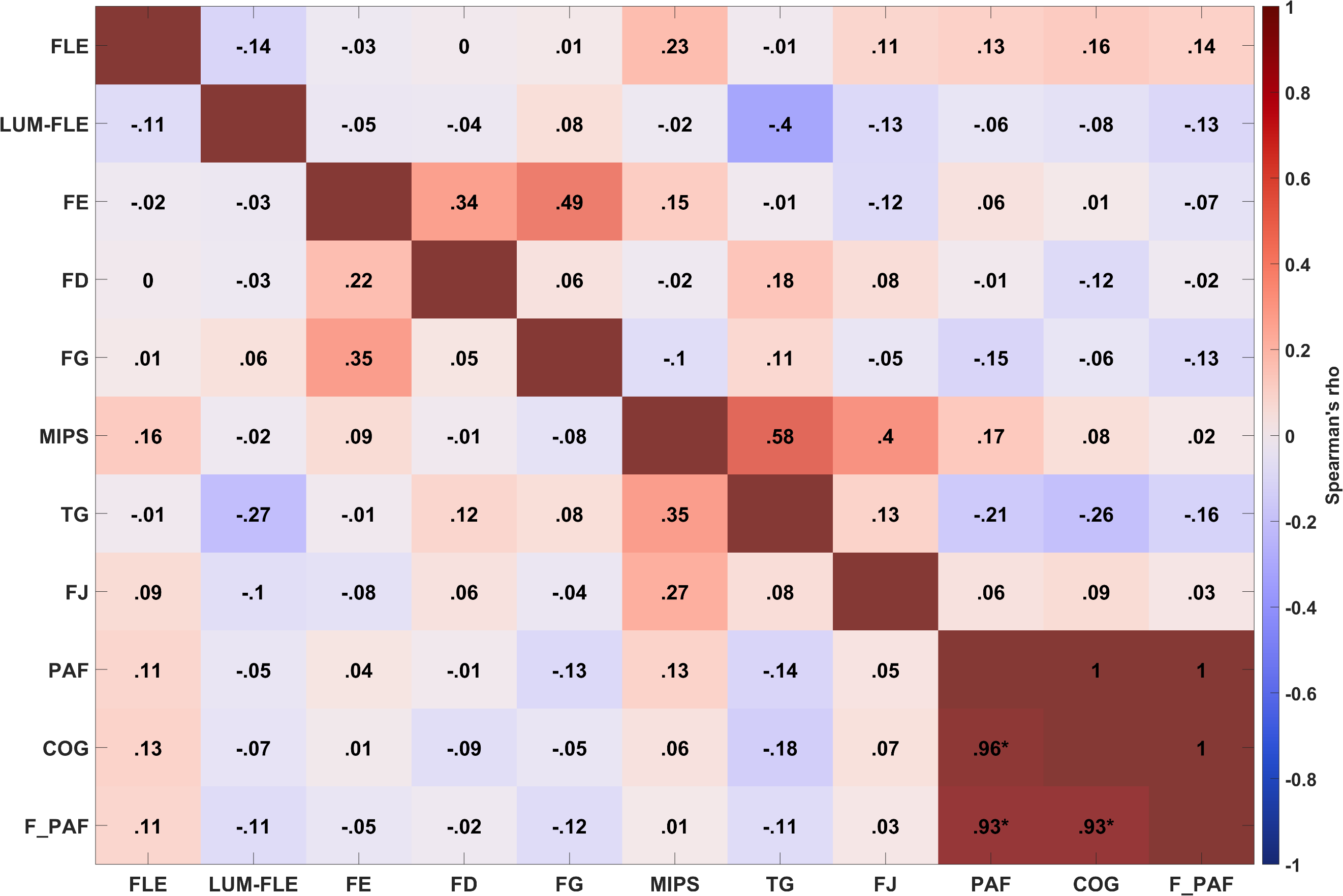


**B**

**A**

|  |  |
| --- | --- |
|  |  |

***Supplementary materials Figure 13.*** Correlations between IAF and the illusions, with IAF calculated with the data from a subset of electrodes (Oz, O1, and O2). Correlations were rounded to two decimal places.  **A.** Show correlations before controlling for age. **B.** Shows correlations after controlling for age. FLE = Flash-lag effect, LUM-FLE = luminance flash-lag effect, FE = Fröhlich effect, FD = flash-drag effect, FG = flash-grab effect, MIPS = motion-induced position shift, TG = twinkle-goes effect, FJ = flash-jump effect, PAF = peak alpha frequency, COG = centre of gravity, F_PAF = FOOOF corrected peak alpha frequency. For the correlations between illusions including all parietal-occipital electrodes, see Figure 4 in main text. Disattenuated correlations are presented above the diagonal red line.

| **Electrodes O1, Oz, O2** | | | | | | | | | | | |  | |
| --- | --- | --- | --- | --- | --- | --- | --- | --- | --- | --- | --- | --- | --- |
|  | **FLE** | **Lum-FLE** | **Fröhlich** | **FD** | **FG** | **MIPS** | **TG** | **FJ** | **PAF** | **COG** | **F_PAF** | |  |
| **FLE** |  | [-0.41, 0.19] | [-0.33, 0.26] | [-0.38, 0.15] | [-0.33, 0.27] | [-0.13, 0.41] | [-0.29, 0.28] | [-0.16, 0.42] | [-0.13, 0.46] | [-0.07, 0.48] | [-0.07, 0.46] | |  |
| **Lum-FLE** | [-0.38, 0.18] |  | [-0.30, 0.28] | [-0.30, 0.30] | [-0.23, 0.35] | [-0.33, 0.31] | [-0.51, 0.005] | [-0.38, 0.19] | [-0.35, 0.25] | [-0.35, 0.21] | [-0.40, 0.21] | |  |
| **Fröhlich** | [-0.30, 0.29] | [-0.31, 0.27] |  | [-0.04, 0.45] | **[0.06, 0.60]** | [-0.16, 0.32] | [-0.26, 0.29] | [-0.34, 0.17] | [-0.20, 0.32] | [-0.24, 0.29] | [-0.29, 0.23] | |  |
| **FD** | [-0.27, 0.27] | [-0.32, 0.27] | [-0.03, 0.45] |  | [-0.24, 0.25] | [-0.29, 0.25] | [-0.13, 0.43] | [-0.13, 0.40] | [-0.17, 0.32] | [-0.24, 0.24] | [-0.20, 0.29] | |  |
| **FG** | [-0.29, 0.29] | [-0.23, 0.33] | **[0.08, 0.58]** | [-0.18, 0.29] |  | [-0.36, 0.17] | [-0.22, 0.37] | [-0.27, 0.26] | [-0.37, 0.23] | [-0.31, 0.29] | [-0.38, 0.20] | |  |
| **MIPS** | [-0.11, 0.4] | [-0.31, 0.27] | [-0.19, 0.32] | [-0.28, 0.30] | [-0.33, 0.23] |  | **[0.1, 0.57]** | [-0.005, 0.53] | [-0.12, 0.40] | [-0.20, 0.31] | [-0.22, 0.28] | |  |
| **TG** | [-0.28, 0.3] | [-0.52, 0.005] | [-0.27, 0.25] | [-0.17, 0.39] | [-0.23, 0.37] | **[0.09, 0.55]** |  | [-0.18, 0.33] | [-0.42, 0.12] | [-0.46, 0.08] | [-0.41, 0.15] | |  |
| **FJ** | [-0.20, 0.38] | [-0.37, 0.19] | [-0.33, 0.19] | [-0.20, 0.31] | [-0.30, 0.20] | [-0.003, 0.54] | [-0.19, 0.34] |  | [-0.27, 0.27] | [-0.24, 0.29] | [-0.26, 0.26] | |  |
| **PAF** | [-0.2, 0.40] | [-0.34, 0.32] | [-0.21, 0.32] | [-0.24, 0.24] | [-0.38, 0.16] | [-0.13, 0.37] | [-0.40, 0.12] | [-0.21, 0.3] |  | **[0.89, 0.98]** | **[0.81, 0.97]** | |  |
| **COG** | [-0.19, 0.43] | [-0.36, 0.25] | [-0.25, 0.29] | [-0.30, 0.16] | [-0.31, 0.23] | [-0.19, 0.31] | [-0.42, 0.11] | [-0.19, 0.31] | **[0.90, 0.98]** |  | **[0.84, 0.97]** | |  |
| **F_PAF** | [-0.21, 0.42] | [-0.39, 0.24] | [-0.30, 0.22] | [-0.27, 0.22] | [-0.38, 0.19] | [-0.24, 0.26] | [-0.36, 0.2] | [-0.21, 0.28] | **[0.81, 0.97]** | **[0.85, 0.97]** |  | |  |

***Supplementary materials Table 3.*** Bootstrapped confidence intervals for the correlations between illusions and IAF (IAF), when IAF is calculated only using the data from electrodes O1, Oz, and O2. Calculated using 95% bias-corrected and accelerated bootstrapping (*N* = 1000). Confidence intervals that do not contain zero are shown in **bold red font**. The blue cells show the confidence intervals for correlations not controlling for age. The red cells show the confidence intervals for correlations controlling for age.

***Supplementary materials Figure 14.*** Correlations between the motion-position illusions using the aggregate sample.


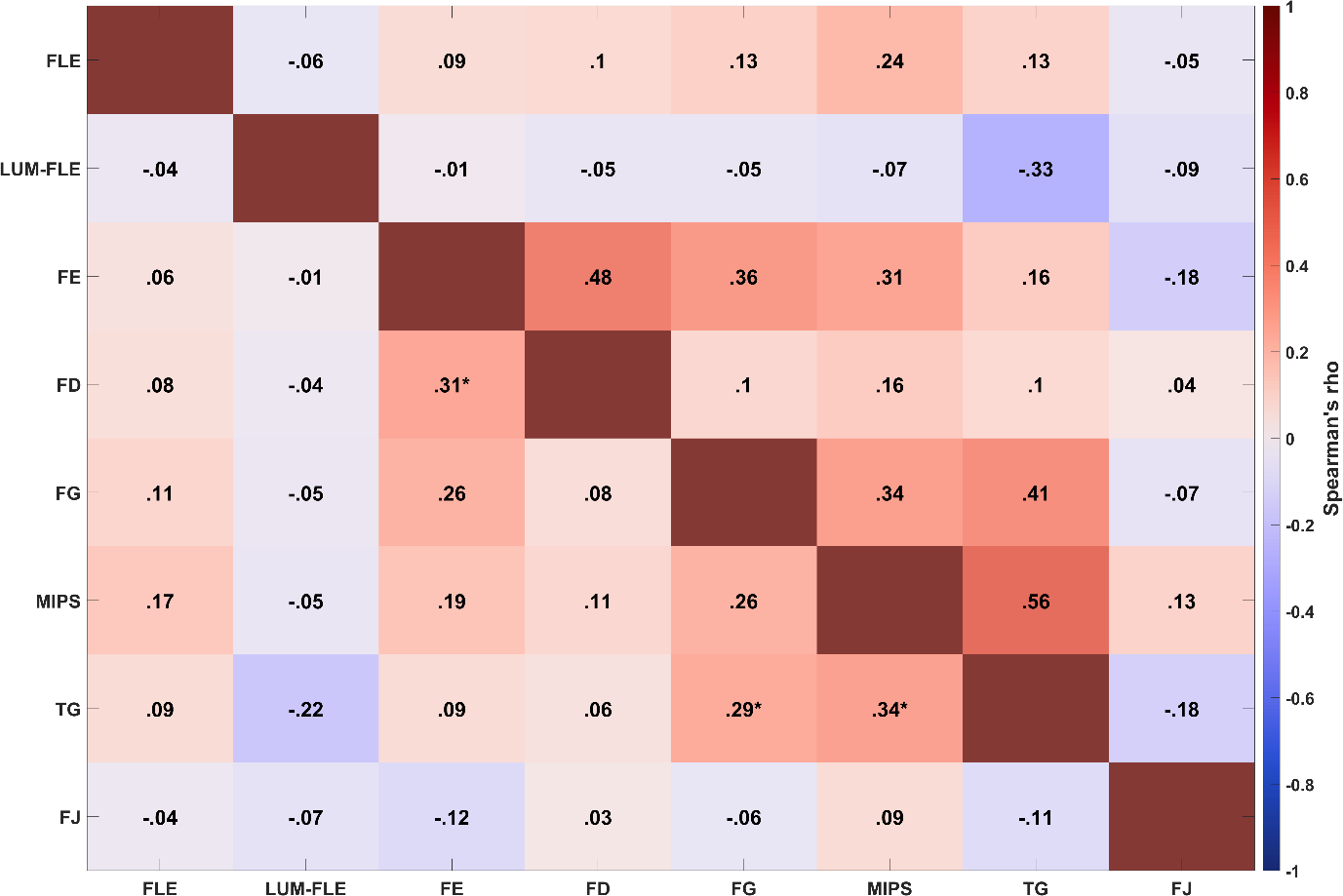


The aggregate sample comprised 149 participants, 106 of which completed two sessions of the illusions in Cottier et al. (2023). We will refer to the participants from Cottier et al. (2023) as old participants. The sample size for each illusion in this correlation matrix comprised: 122 participants  (85 old participants) completed the flash-lag effect (FLE), 117 (83 old) completed the luminance flash-lag effect (LUM-FLE), 125 (82 old) the Fröhlich effect (FE), 146 (103 old) completed the flash-drag effect (FD), 138 (99 old) completed the flash-grab effect (FG), 146 (104 old) completed the motion-induced position shift (MIPS), 136 (96 old) completed the twinkle-goes effect, and 138 (97 old) completed the flash-jump effect (FJ). Disattenuated correlations are presented above the diagonal red line. The p-values for the correlations are presented below, in Supplementary materials table 4.

| **Illusions (*N*)** | FLE | LUM-FLE | Fröhlich | FD | FG | MIPS | TG |
| --- | --- | --- | --- | --- | --- | --- | --- |
| FLE (122) |  |  |  |  |  |  |  |
| Lum-FLE (117*)* | .6674 |  |  |  |  |  |  |
| Fröhlich *(125)* | .5521 | .9469 |  |  |  |  |  |
| FD (*146)* | .4150 | .7059 | **.0005** |  |  |  |  |
| FG *(138)* | .2348 | .6353 | .0039 | .3442 |  |  |  |
| MIPS *(146)* | .0662 | .5915 | .0327 | .1969 | .0021 |  |  |
| TG *(136)* | .3584 | .0201 | .3267 | .4894 | **.0009** | **.0001** |  |
| FJ (138) | .6808 | .4661 | .1976 | .7207 | .5216 | .2998 | .1979 |

***Supplementary materials Table 4.*** P-values for the correlation analysis between the illusions, using the aggregate sample. The aggregate sample comprised the illusion magnitudes from the present study, and the illusion magnitude averaged across sessions from Cottier et al. (2023). Statistically significant p values after Bonferroni-Holm correction for multiple comparisons are in **bold.**

**Discussion of the aggregate sample correlations:**

As mentioned in the main text, 149 participants were included in the aggregate sample. 43 unique participants from the present study, and 106 from Cottier et al. (2023). Because participants in Cottier et al. (2023) completed the illusions twice across two separate sessions, we averaged their illusory effects across sessions. One of the critiques of Cottier et al. (2023), is that the conservative Bonferroni correction used to control for multiple comparisons might not have detected some true correlations. To address this possibility, we controlled for multiple comparisons by conducting the less conservative Bonferroni-Holm correction.

The correlation analysis with this aggregate sample replicated all but one of the observations of Cottier et al. (2023). Consistent with Cottier et al. (2023), we observed the same two correlated clusters of illusions. One cluster comprising the flash-drag effect and Fröhlich effect, and another cluster comprising the twinkle-goes effect, motion-induced position shift, and the flash-grab effect. The only difference to Cottier et al. (2023), is that the correlation between FG and MIPS did not reach significance (p=0.0021; corrected alpha 0.002). Overall, it remains unclear why were not able to replicate the findings of Cottier et al. (2023). There are two possibilities for future research to explore; firstly, the effect was smaller than initially reported. Secondly, the effect was not detected due to increased measurement noise in the estimate of the illusion magnitude.
